# Supplementary figures and images for: MESSAR: Automated recommendation of metabolite substructures from tandem mass spectra
Source: PLoS One. 2020 Jan 16;15(1):e0226770. doi: 10.1371/journal.pone.0226770 (PMC6964822; doi:10.1371/journal.pone.0226770)

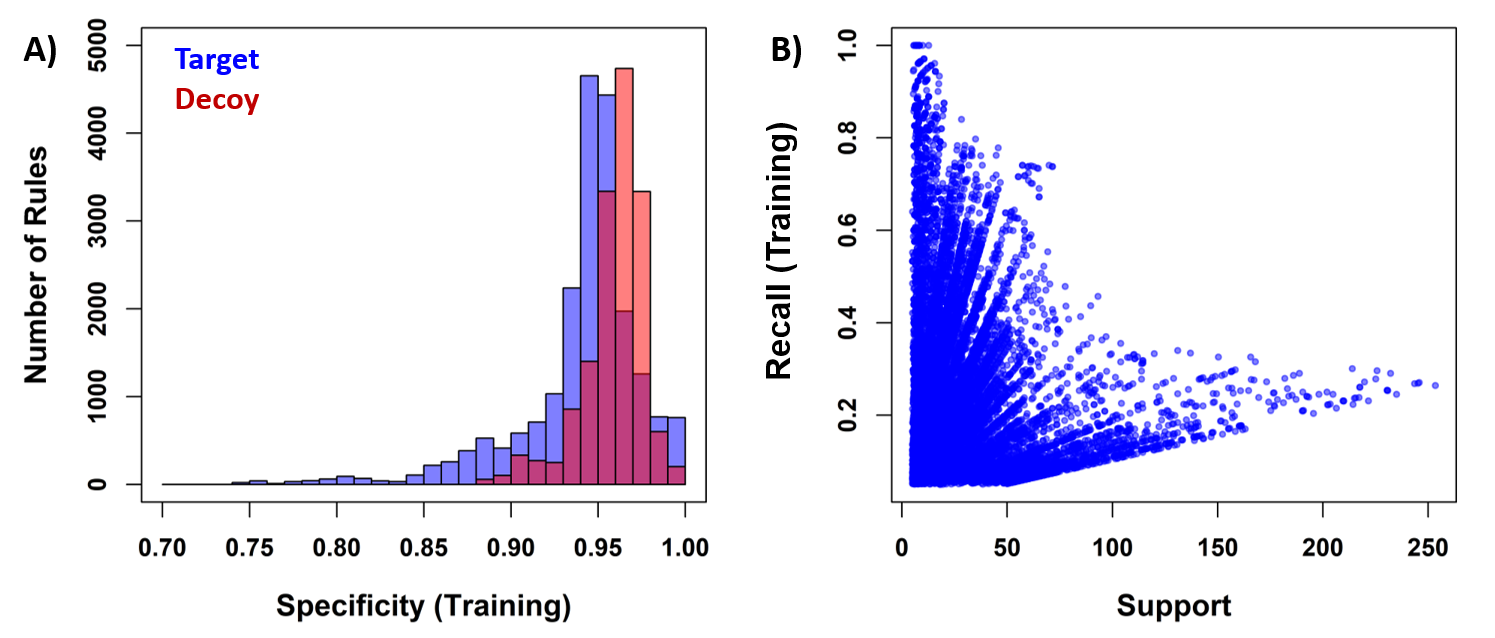

Supplement: S1 Fig — A) Distribution of target/decoy rule specificity. B) Dependency between recall of target rules and support. (PNG) [file pone.0226770.s002.png]

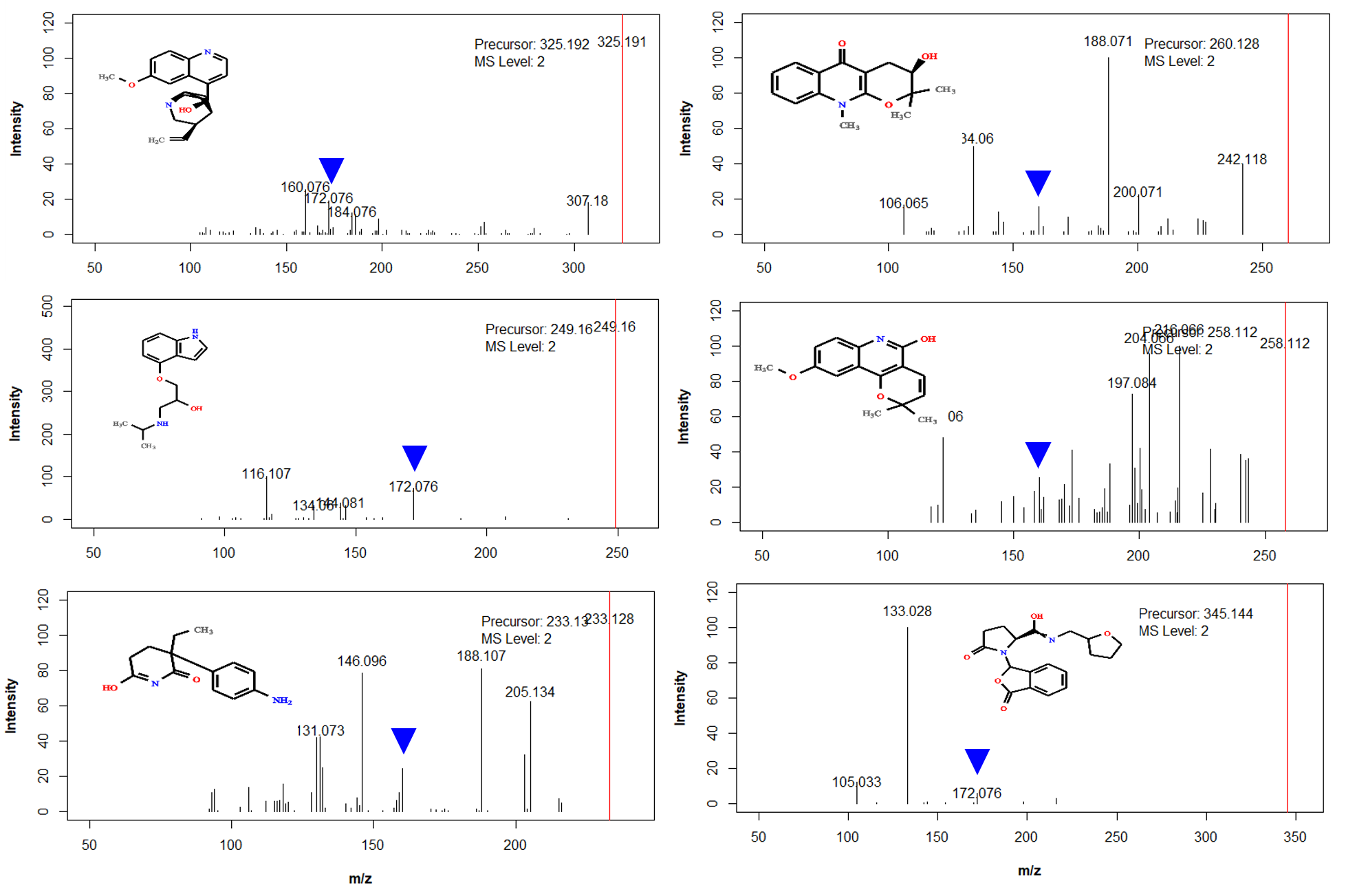

Supplement: S2 Fig — The peak 172.075 is present in all examples, while the underlying training molecules were structurally similar—little or no substructure difference was observed. (PNG) [file pone.0226770.s003.png]

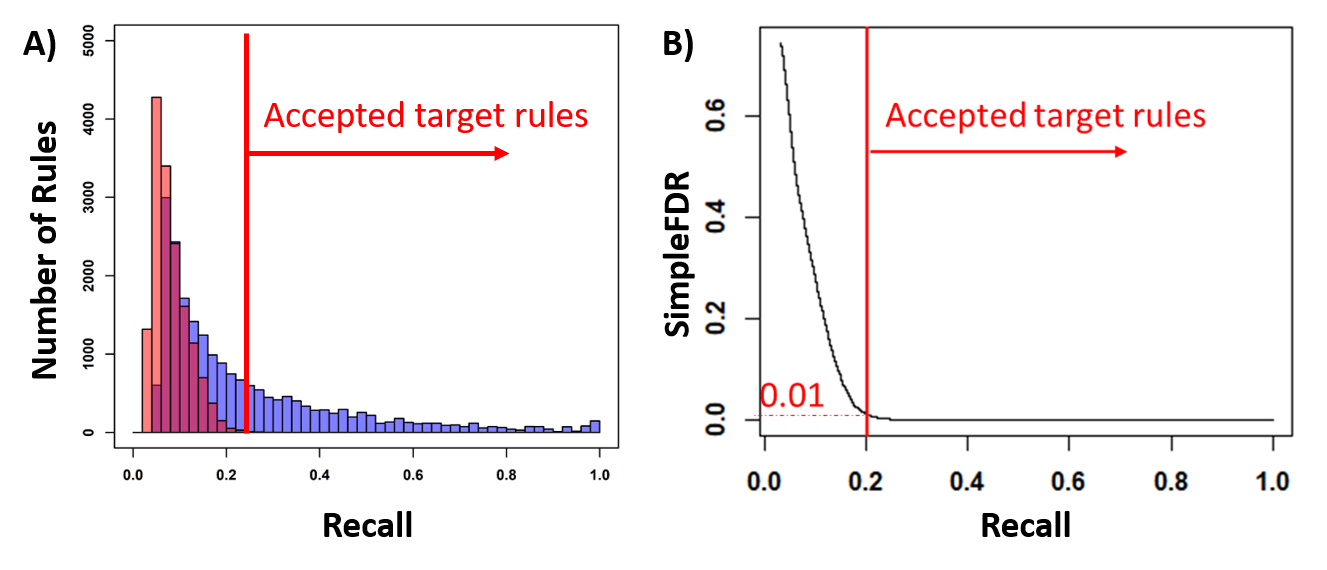

Supplement: S3 Fig — A) Recall distribution of target and decoy rules. B) FDR score of rules as a function recall. The red vertical line in both plots indicates the recall threshold estimated for 1% FDR. (PNG) [file pone.0226770.s004.png]
