# Supplementary material for: MESSAR: Automated recommendation of metabolite substructures from tandem mass spectra
Source: PLoS One. 2020 Jan 16;15(1):e0226770. doi: 10.1371/journal.pone.0226770 (PMC6964822; doi:10.1371/journal.pone.0226770)
Supplement: S3 Table — (PDF) [file pone.0226770.s007.pdf]

Table S3 Substructure prediction by MESSAR, and MS2LDA for 185 challenge spectra along with the ground-truth. The external expert decided for each challenge the meaningful and relevant substructure(s) compared to the ground-truth (without knowing the name of software used to generate each substructure). The selected substructures were inside blue rectangles. We report for each challenge the tool(s) that generate the selected substructure(s).

Example:

ID	MESSAR		MS2LDA	Ground-truth	Selected method
38	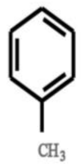 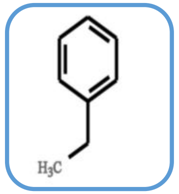 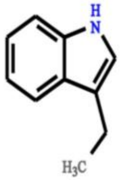	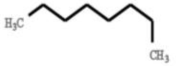 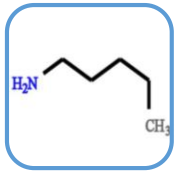 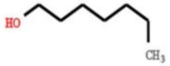	CO loss - indicative for presence of tone/aldehyde/lactor group (C=O)	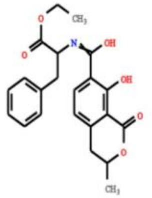	MESSAR & CSI: FingerID

1	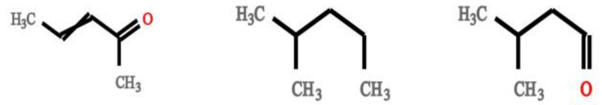	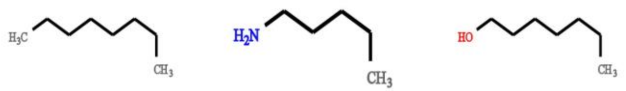	None	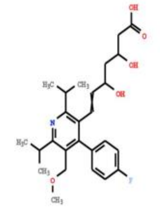	None
2	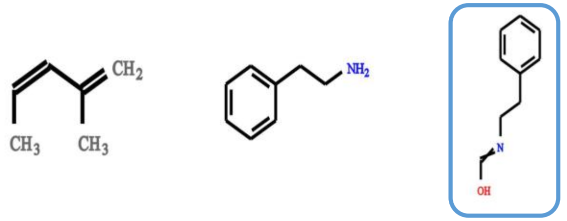	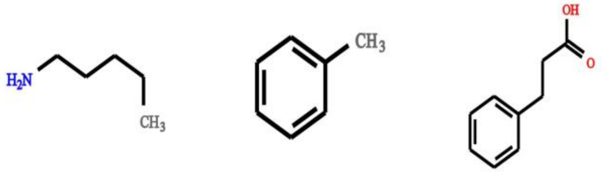	Fragment indicative for aromatic compounds related to methylbenzene substructure (C_7H_7 fragment)	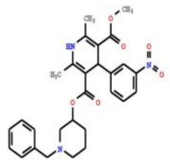	MESSAR
3	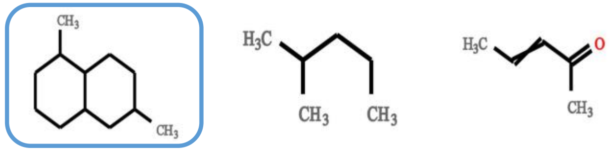	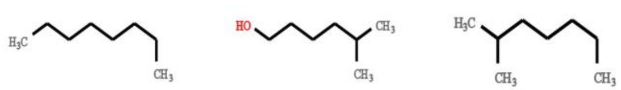	Steroid core related ($C_{18}H_{21}$ and smaller fragments thereof - with $C_{12}H_{13}$, $C_{11}H_{11}$, and $C_{11}H_{13}$ most probable)	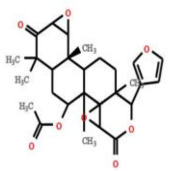	MESSAR & MS2LDA
4	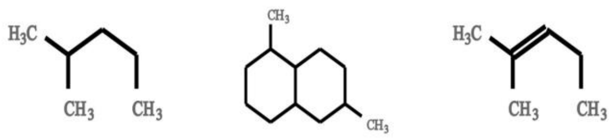	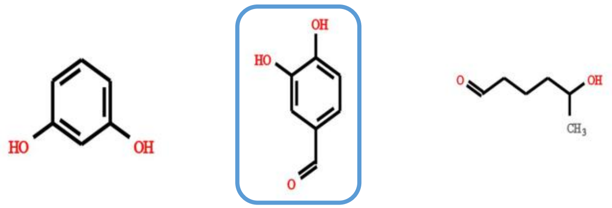	CO loss - indicative for presence of etone/aldehyde/lactor group (C=O)	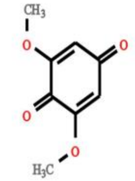	CSI: FingerID
5	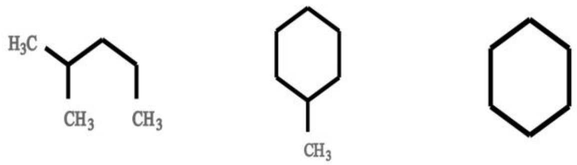	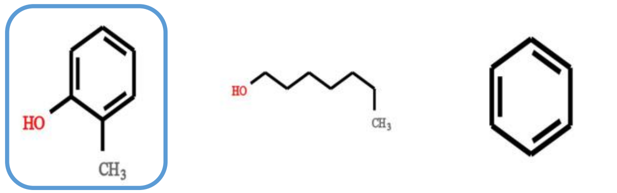	None	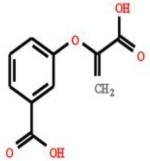	CSI: FingerID

6	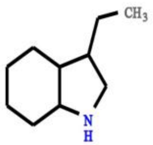	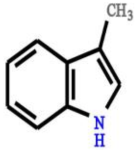	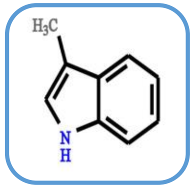	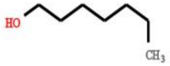	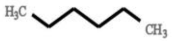	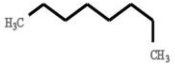	'.3.1.0~2,7~]trideca-2 substructure	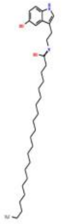	MESSAR
7	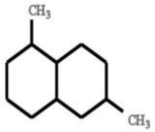	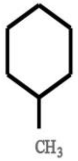	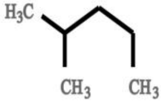	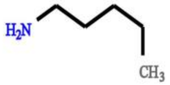	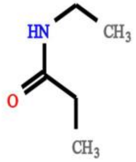	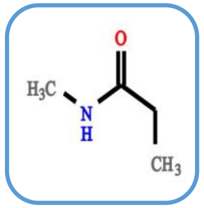	Fragment indicative for aromatic compounds related to methylbenzene substructure (C7H7 fragment)	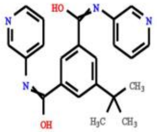	CSI: FingerID
8	None	None	None	None	None	None	None	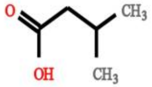	None
9	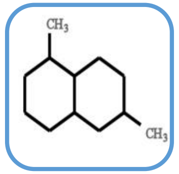	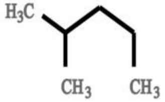	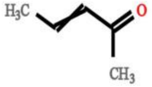	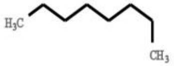	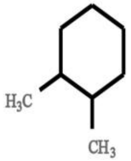	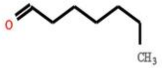	Water loss - indicative of a free hydroxyl group â€“ (in beer often seen in sugary structures)	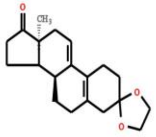	MESSAR
10	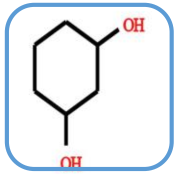	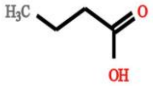	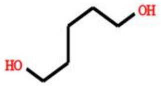	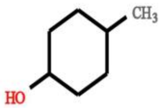	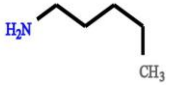	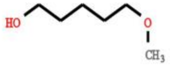	None	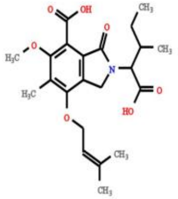	MESSAR

11	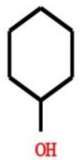	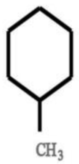	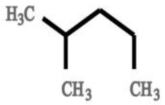	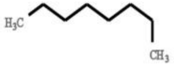	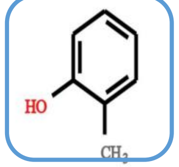	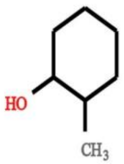	None	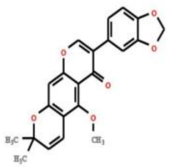	CSI: FingerID
12	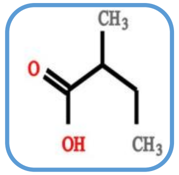	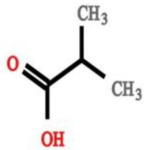	None	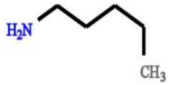	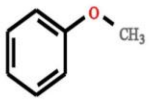	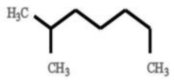	None	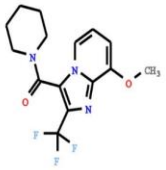	MESSAR
13	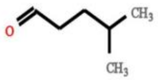	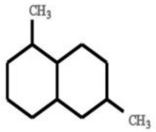	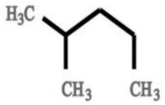	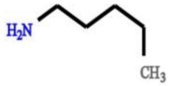	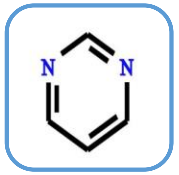	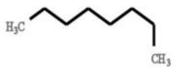	None	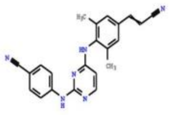	CSI: FingerID
14	None	None	None	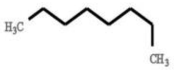	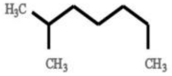	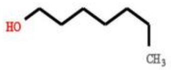	1-keto-chlorobenzene substructure	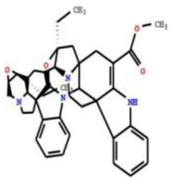	None
15	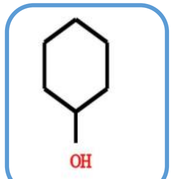	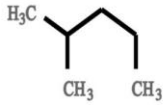	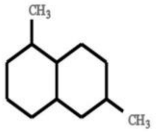	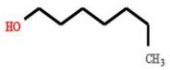	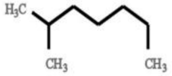	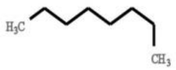	Fragments indicative for namic/hydroxycinnar acid substructure	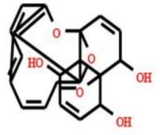	MESSAR

16	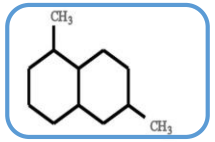	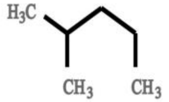	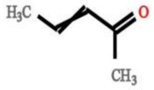	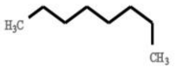	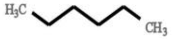	None	None	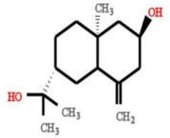	MESSAR
17	None	None	None	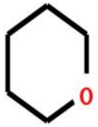	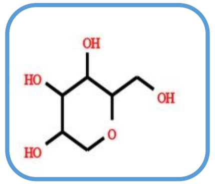	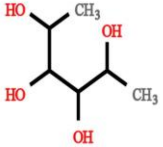	benzene substructure [ClassyFire - Relevant terms - Substituents: Methoxybenzene, Phenoxy compounds - Taxa: O-methylated	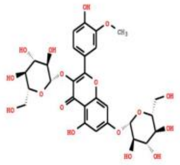	CSI: FingerID &MS2LDA
18	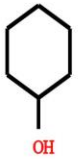	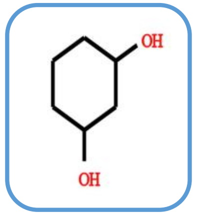	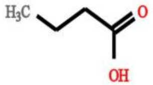	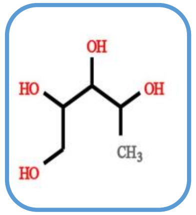	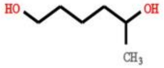	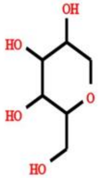	phthalate substructure	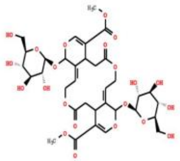	MESSAR & CSI: FingerID
19	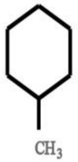	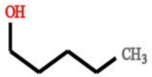	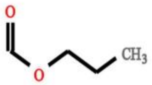	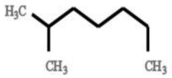	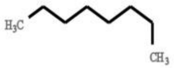	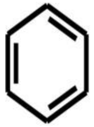	phenyl)-2-oxo-2H-chr relared substructure (or isomeric variants)	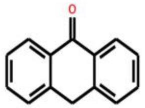	None
20	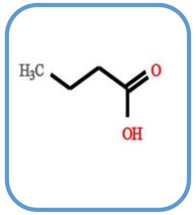	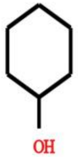	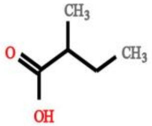	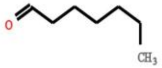	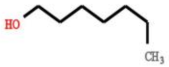	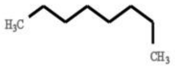	Fragments indicative for namic/hydroxycinna acid substructure	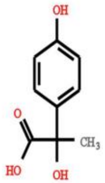	MESSAR &MS2LDA

21	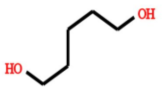	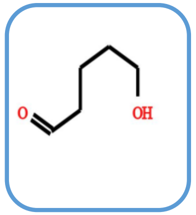	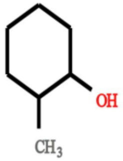	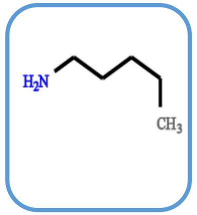	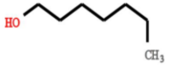	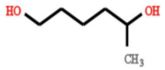	None	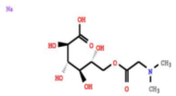	MESSAR & CSI: FingerID
22	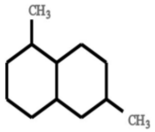	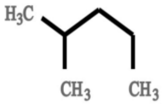	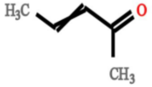	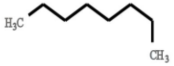	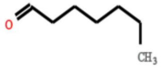	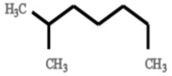	None	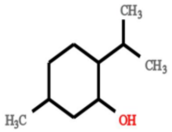	None
23	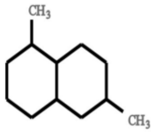	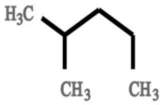	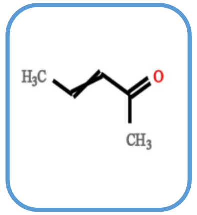	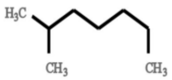	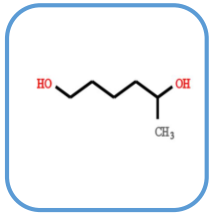	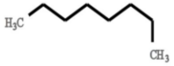	Steroid core related (C18H21 and smaller fragments thereof - with C12H13, C11H11, and C11H13 most probable)	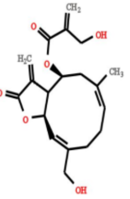	MESSAR & CSI: FingerID
24	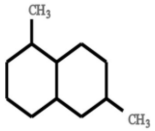	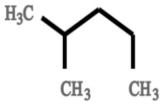	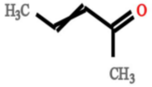	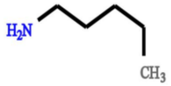	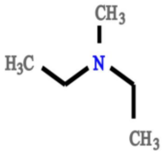	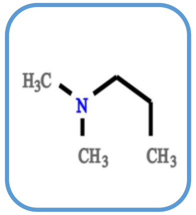	None	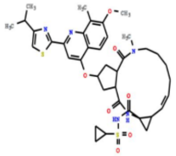	CSI: FingerID
25	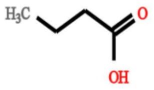	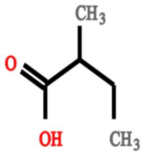	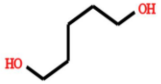	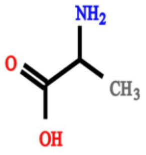	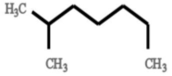	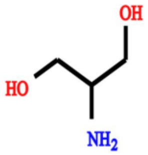	None	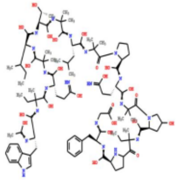	None

26	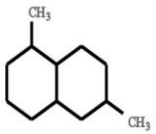	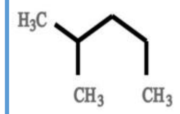	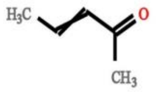	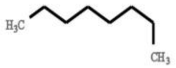	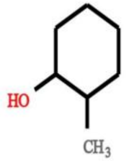	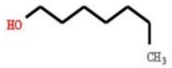	2-oxochromen-7-yl [mainly trimethylated] related substructure	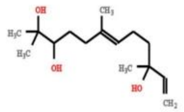	MESSAR
27	None	None	None	None	None	None	cinchonan-9-ol substructure	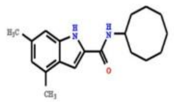	MS2LDA
28	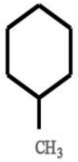	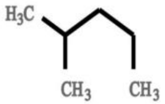	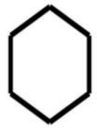	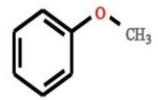	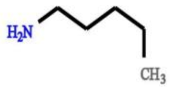	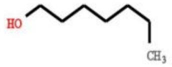	Sterone related	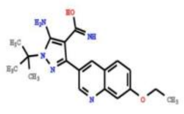	CSI: FingerID
29	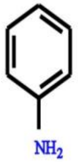	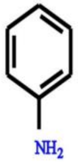	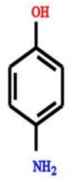	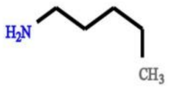	None	None	None	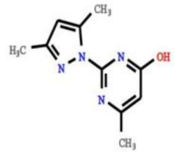	None
30	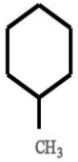	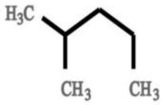	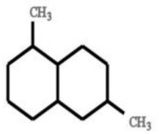	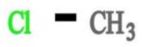	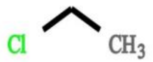	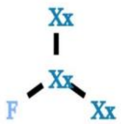	None	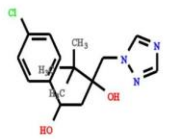	CSI: FingerID

31	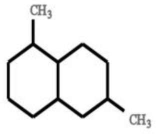	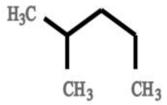	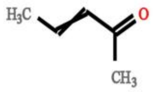	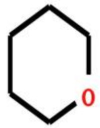	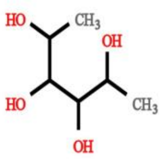	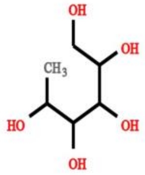	Fragments indicative of a glycosylation â€“ i.e. indicative for a sugar conjugation (in beer often related to glucose)	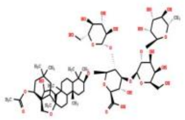	CSI: FingerID &MS2LDA
32	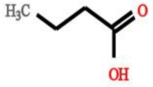	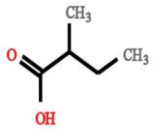	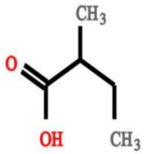	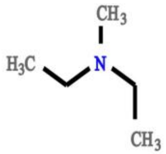	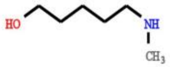	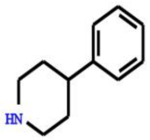	None	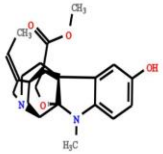	None
33	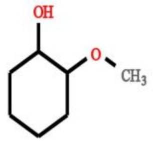	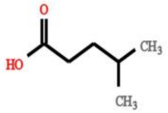	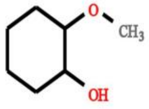	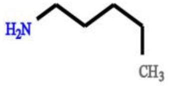	None	None	None	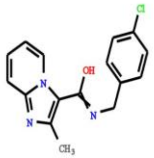	None
34	None	None	None	None	None	None	None	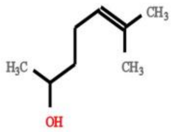	None
35	None	None	None	None	None	None	Aliphatic amine (NH3 loss indicates free NH2 group coupled to aliphatic chain)	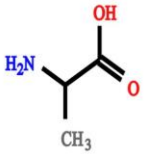	MS2LDA

36	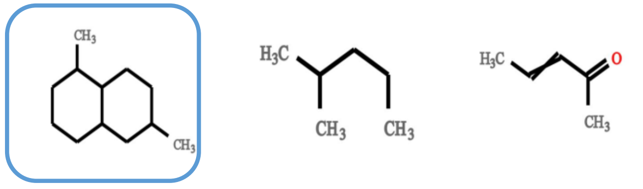	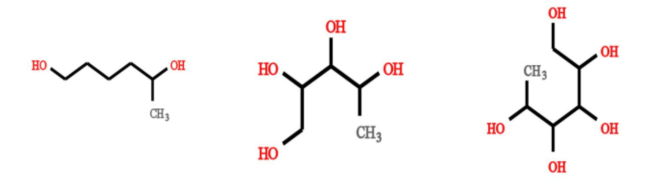	ydroxy-Pregnenolon related fragments (not very specific - indicative for presence of steroid backbone)	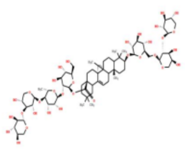	MESSAR &MS2LDA
37	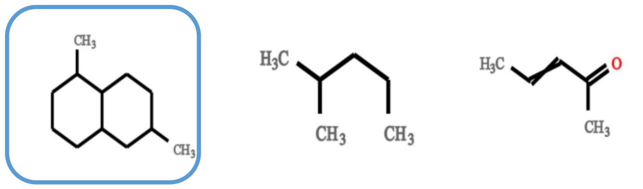	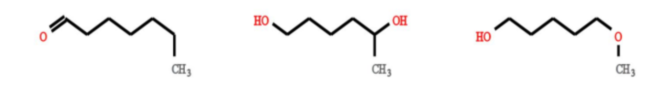	Sterone related	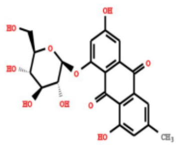	MESSAR &MS2LDA
38	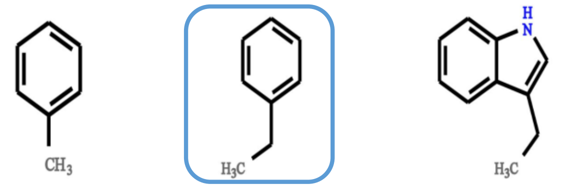	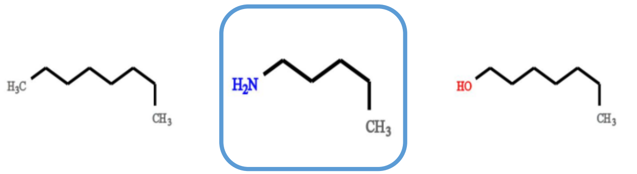	CO loss - indicative for presence of etone/aldehyde/lactor group (C=O)	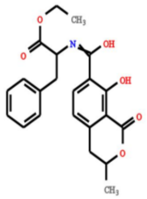	MESSAR
39	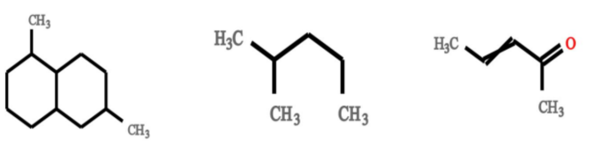	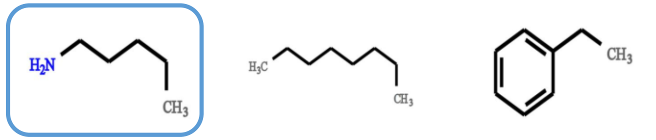	nitrogen containing substructure [C5H12N] (in beer related to Leucine)	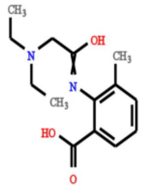	CSI: FingerID &MS2LDA
40	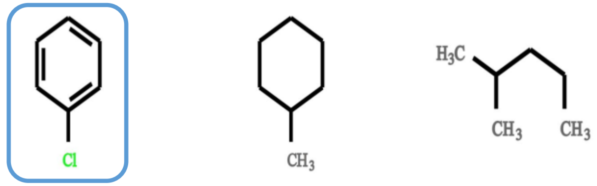	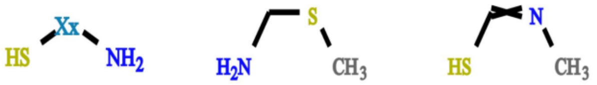	CO loss - indicative for presence of etone/aldehyde/lactor group (C=O)	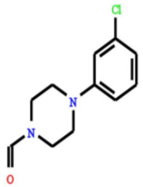	MESSAR

41	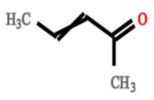	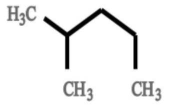	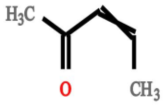	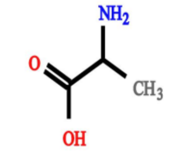	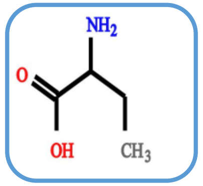	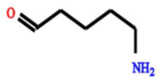	Fragment and loss of [proline-H2O] - indicative for conjugated proline or arginine/ornithine â€“ EF fits	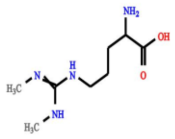	CSI: FingerID & MS2LDA
42	None	None	None	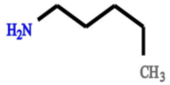	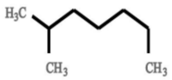	None	[illegible] substructure (or formed after fragmentation event of dihydro analogue) [ClassyFire: Relevant terms - Substituents: Quinoxaline - [illegible]	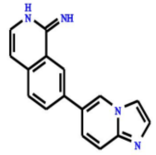	MS2LDA
43	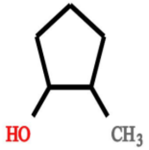	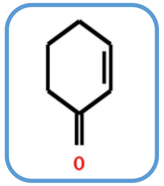	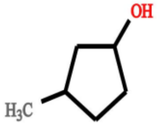	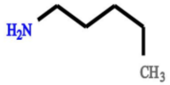	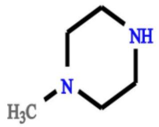	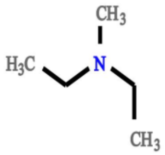	None	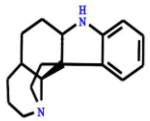	MESSAR
44	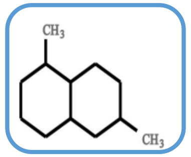	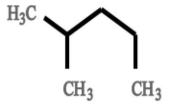	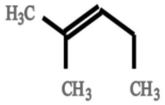	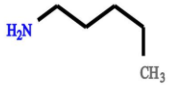	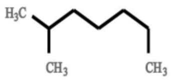	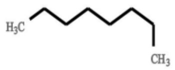	None	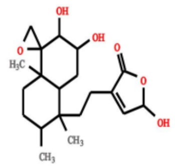	MESSAR
45	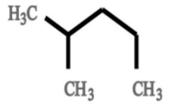	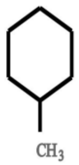	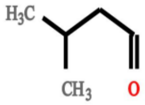	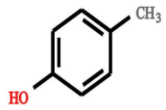	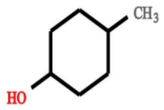	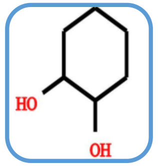	Fragments indicative for ferulic acid based substructure (MzCloud)	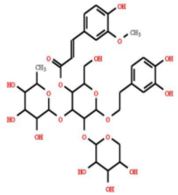	CSI: FingerID & MS2LDA

41	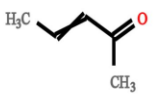	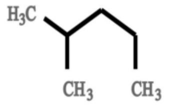	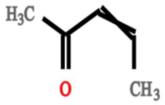	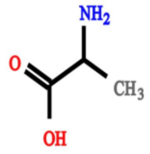	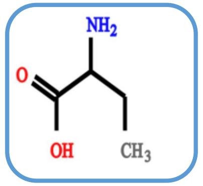	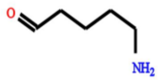	Fragment and loss of [proline-H2O] - indicative for conjugated proline or arginine/ornithine â€“ EF fits	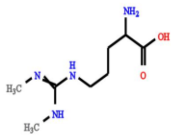	CSI: FingerID & MS2LDA
42	None	None	None	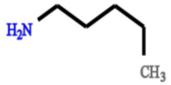	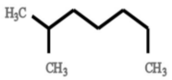	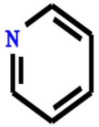	substructure (or formed after fragmentation event of dihydro analogue) [ClassyFire: Relevant terms - Substituents: [illegible]	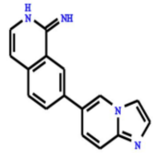	MS2LDA
43	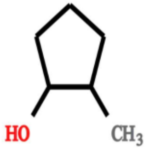	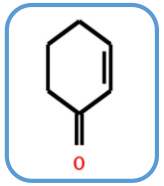	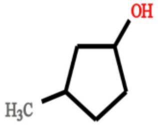	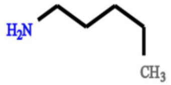	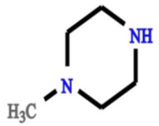	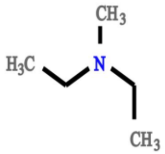	None	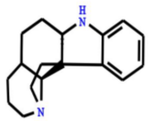	MESSAR
44	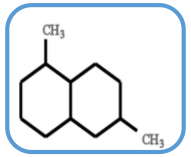	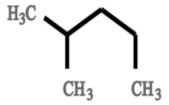	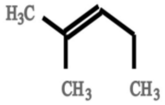	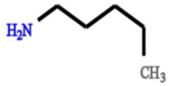	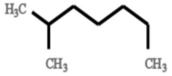	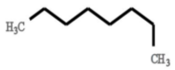	None	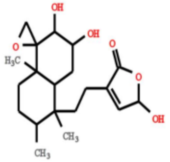	MESSAR
45	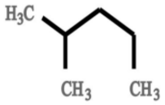	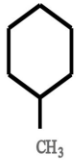	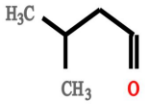	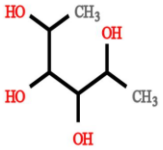	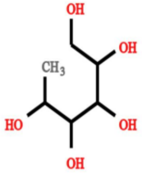	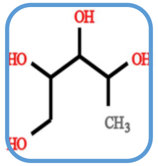	Fragments indicative for ferulic acid based substructure (MzCloud)	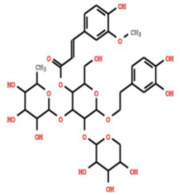	CSI: FingerID & MS2LDA

46	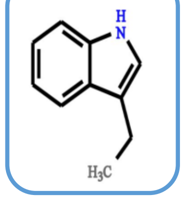	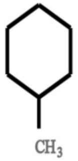	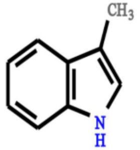	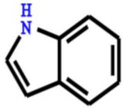	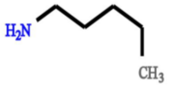	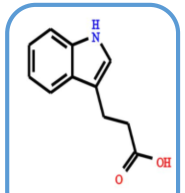	Amine loss - Indicative for free NH2 group in fragmented molecule	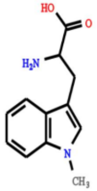	MESSAR & CSI: FingerID
47	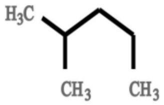	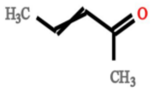	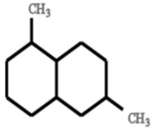	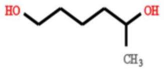	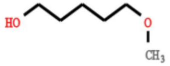	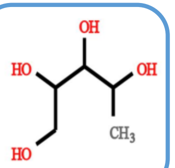	None	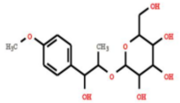	CSI: FingerID
48	None	None	None	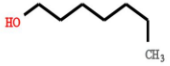	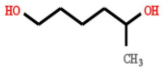	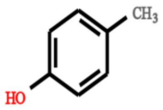	None	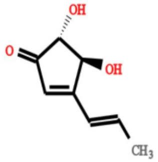	None
49	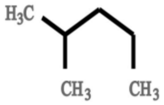	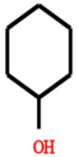	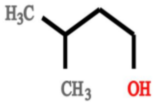	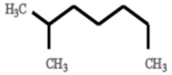	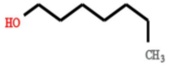	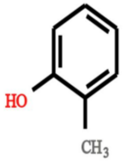	2-oxochromen-7-yl [mainly dimethylated] related substructure	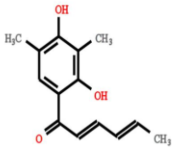	CSI: FingerID
50	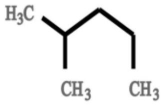	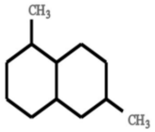	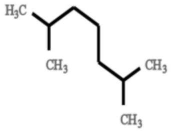	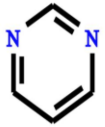	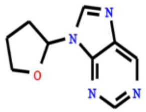	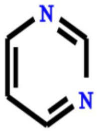	Fragments indicative adenine (C5H6N5) substructure â€“ most prevalent in Beer3	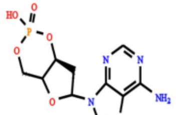	CSI: FingerID

51	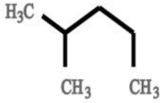	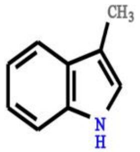	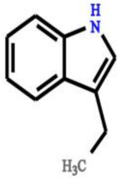	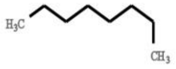	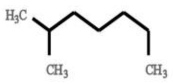	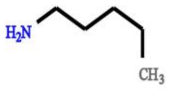	None	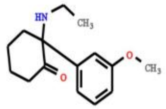	None
52	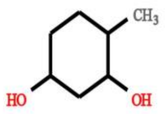	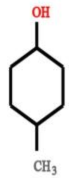	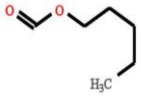	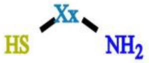	None	None	Small nitrogen containing fragment ion â€“ often proline or ornithine derived â€“ most abundant fragment in beer data	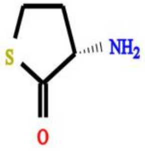	None
53	None	None	None	FH	$F - CH_3$	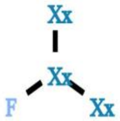	None	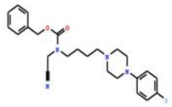	CSI: FingerID
54	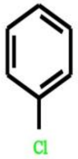	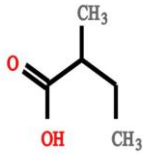	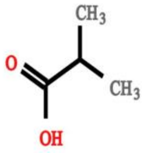	None	None	None	Water loss - indicative of a free hydroxyl group â€“ (in beer often seen in sugary structures)	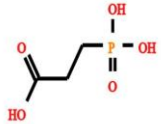	None
55	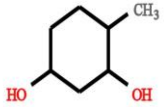	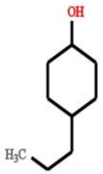	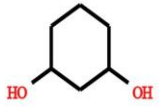	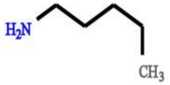	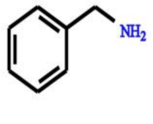	None	nitrogen containing substructure [C5H12N] (in beer related to Leucine)	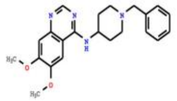	MESSAR & CSI: FingerID

56	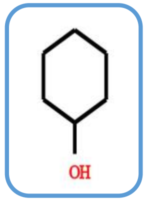	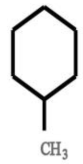	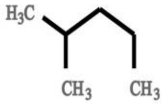	None	None	None	None	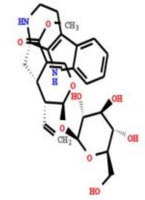	MESSAR
57	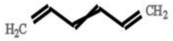	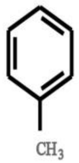	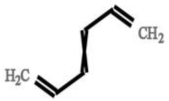	None	None	None	None	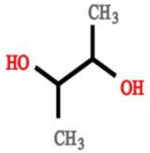	None
58	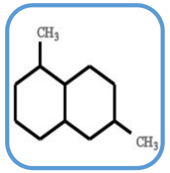	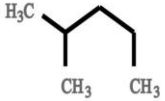	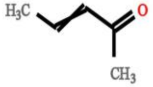	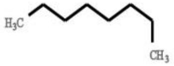	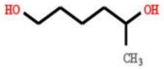	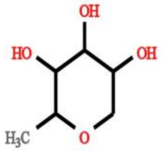	Steroid core related ($C_{18}H_{21}$ and smaller fragments thereof - with $C_{12}H_{13}$, $C_{11}H_{11}$, and $C_{11}H_{13}$ most probable)	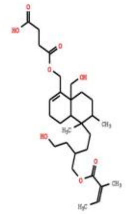	MESSAR & MS2LDA
59	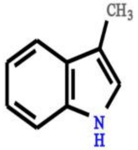	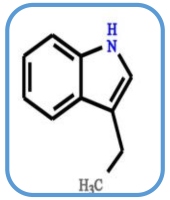	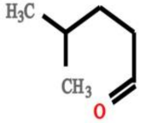	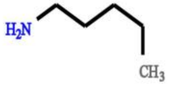	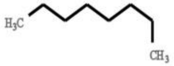	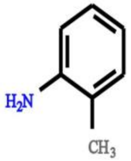	None	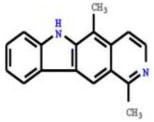	MESSAR
60	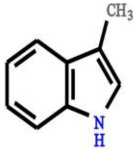	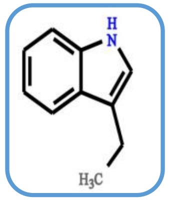	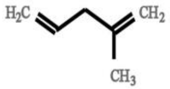	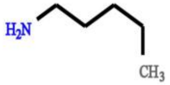	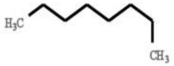	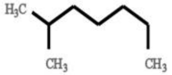	C_4H_8 loss indicative for saturated C4-alkyl substructure (often tert-butyl group or loss from [illegible]0-dihydro-2H,8H-pyr[illegible] substructure)	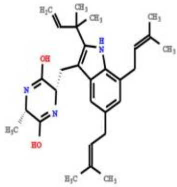	MESSAR

61	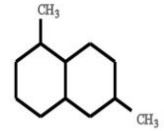	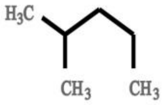	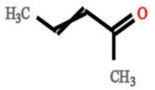	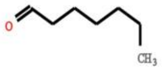	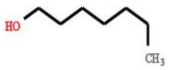	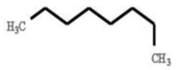	None	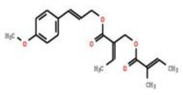	None
62	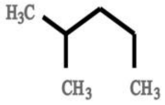	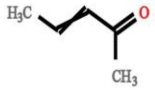	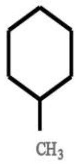	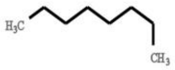	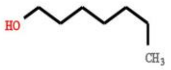	None	None	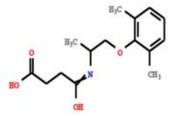	None
63	None	None	None	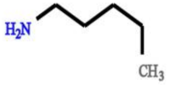	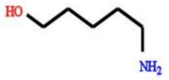	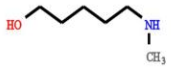	Fragment ions indicative for alkylamine substructure C5H10N (in beer often pipecolic acid [pipecolate])	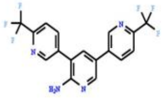	None
64	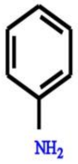	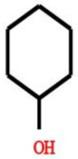	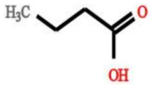	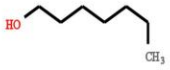	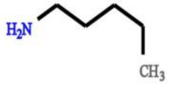	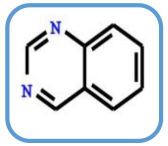	Amine loss - Indicative for free NH2 group in fragmented molecule	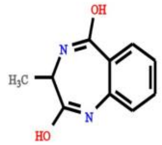	CSI: FingerID
65	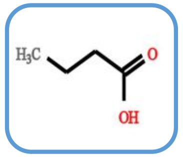	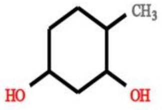	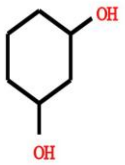	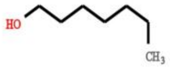	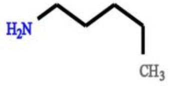	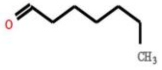	Loss of CH2O2 - indicative for underivatized carboxilic acid group	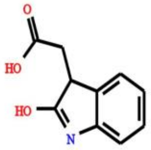	MESSAR

66	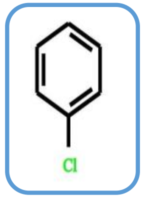	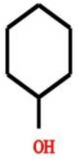	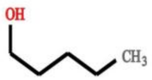	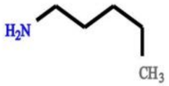	None	None	indicative of piperazine substructure (or related N-containing ring structures for C2H5N loss only)	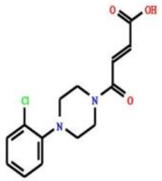	MESSAR & MS2LDA
67	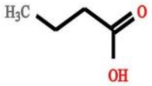	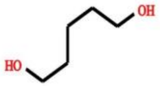	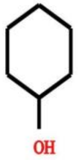	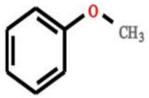	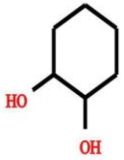	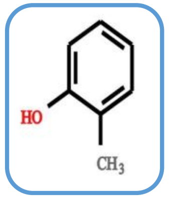	Methoxylated benzene substructure [ClassyFire - Relevant terms - Substituents: Methoxybenzene, Phenoxy compounds - Taxa: O-methylated flavonoids]	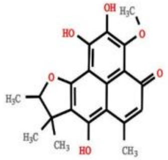	CSI: FingerID
68	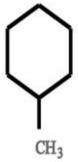	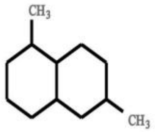	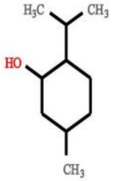	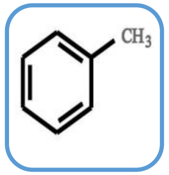	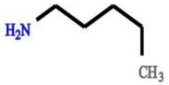	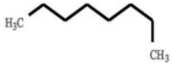	Fragment indicative for aromatic compounds related to methylbenzene substructure (C7H7 fragment)	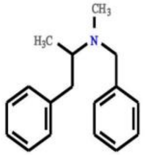	CSI: FingerID
69	None	None	None	None	None	None	None	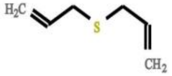	None
70	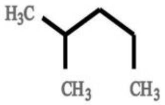	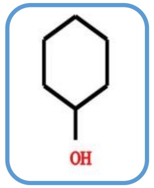	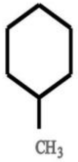	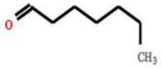	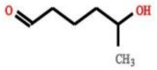	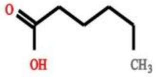	CO loss - indicative for presence of ketone/aldehyde/lactone group (C=O)	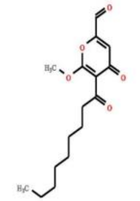	MESSAR

71	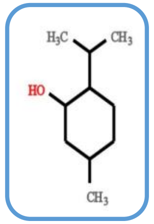	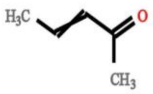	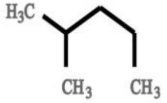	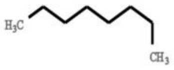	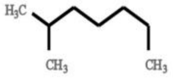	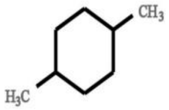	None	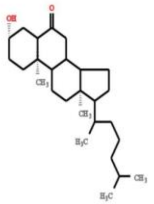	MESSAR
72	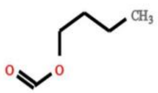	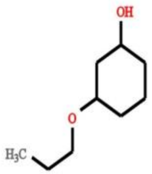	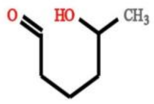	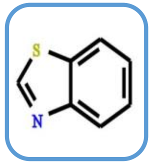	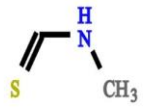	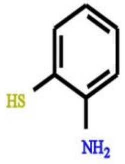	None	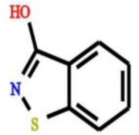	CSI: FingerID
73	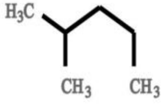	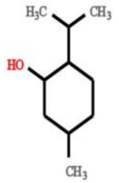	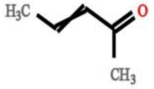	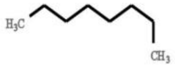	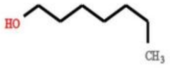	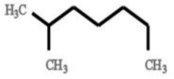	None	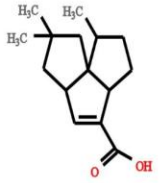	None
74	None	None	None	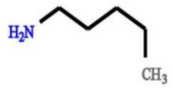	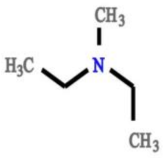	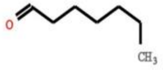	None	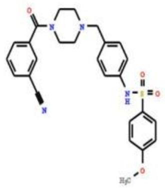	None
75	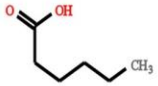	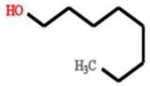	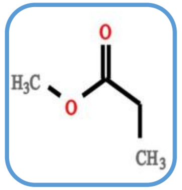	None	None	None	None	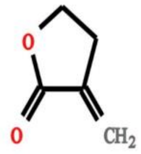	MESSAR

76	None	None	None	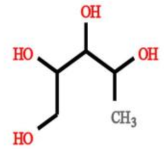	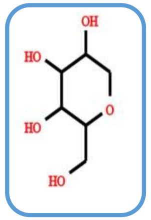	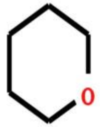	[Pentose (C5-sugar)-H2O] related loss â€“ indicative for conjugated pentose sugar - EF fits	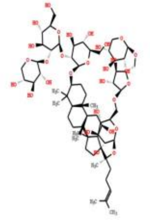	&MS2LDA
77	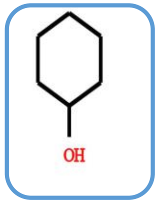	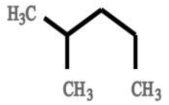	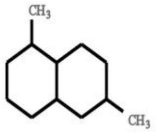	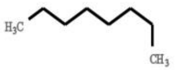	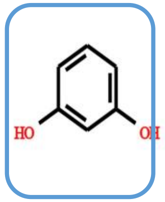	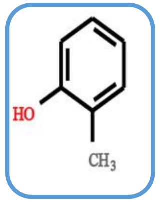	Fragments indicative for tyrosine related substructure (MzCloud)	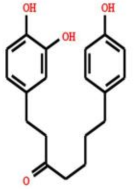	MESSAR<br>& CSI: FingerID
78	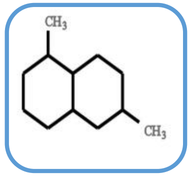	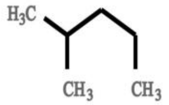	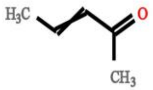	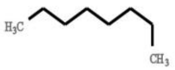	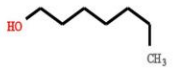	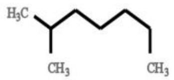	Sterone related	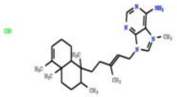	MESSAR<br>&MS2LDA
79	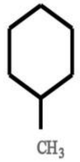	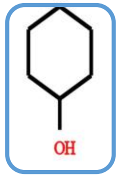	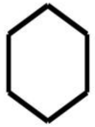	None	None	None	None	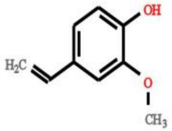	MESSAR
80	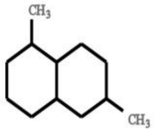	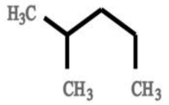	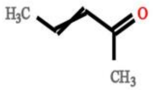	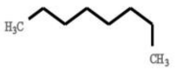	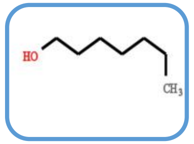	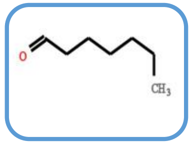	Steroid core related (C18H21 and smaller fragments thereof - with C12H13, C11H11, and C11H13 most probable)	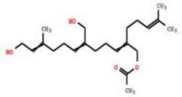	CSI: FingerID

81	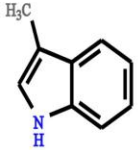	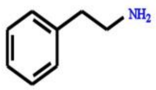	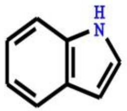	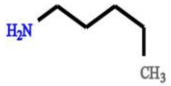	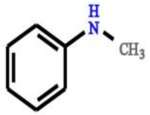	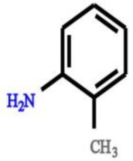	None	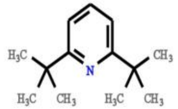	None
82	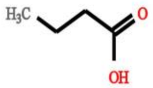	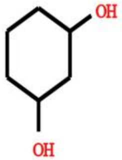	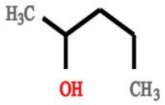	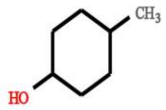	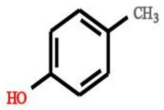	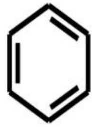	None	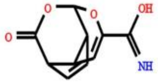	None
83	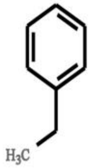	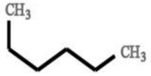	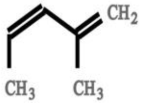	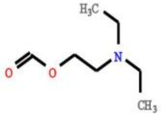	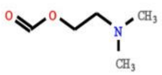	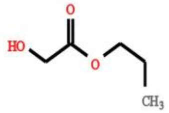	Loss of CH2O2 - indicative for underivatized carboxilic acid group	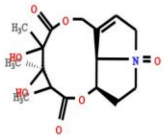	CSI: FingerID
84	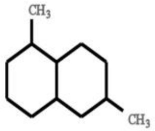	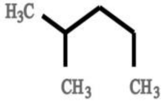	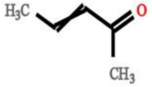	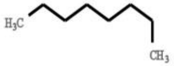	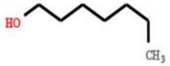	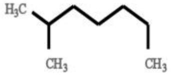	Sterone related	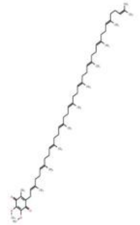	MESSAR & MS2LDA
85	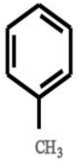	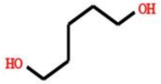	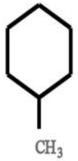	None	None	None	Fragments indicative for ethylphenol substructure (i.e. resulting from Tyramine â€“ MzCloud)	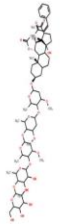	None

86	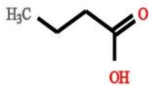	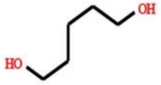	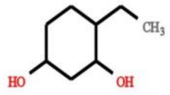	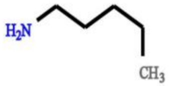	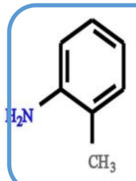	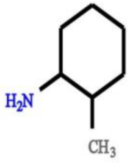	None	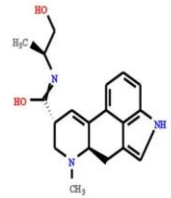	CSI: FingerID
87	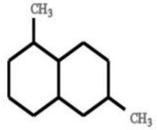	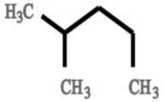	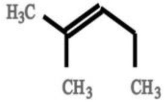	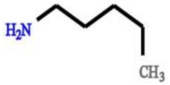	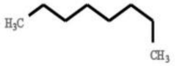	None	Water loss - indicative of a free hydroxyl group â€“ (in beer often seen in sugary structures)	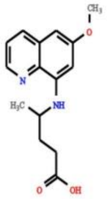	None
88	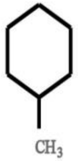	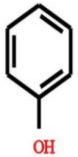	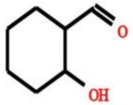	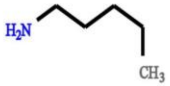	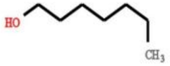	None	None	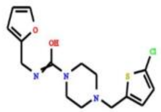	None
89	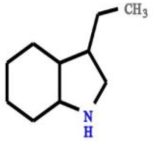	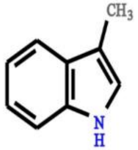	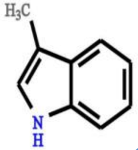	$Cl - CH_3$	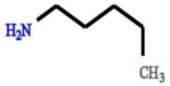	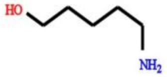	ahydro-1H-imidazo[1 substructure	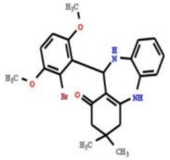	MESSAR
90	None	None	None	None	None	None	None	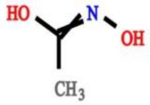	None

91	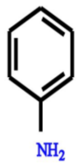	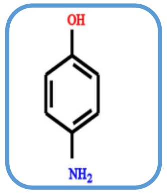	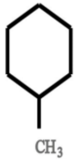	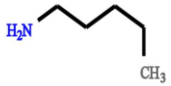	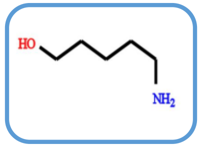	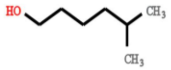	Loss of CH2O2 - indicative for underivatized carboxilic acid group	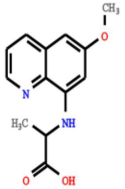	MESSAR & CSI: FingerID
92	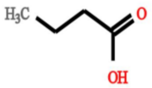	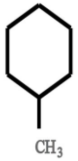	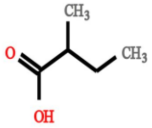	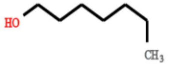	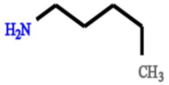	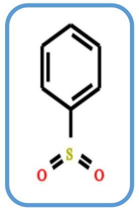	sopropyl/propylamine substructure (loss based) or isopropyl/propyl side chain	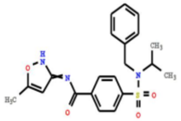	CSI: FingerID
93	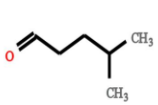	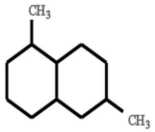	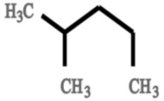	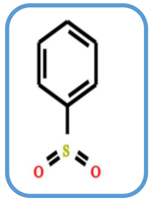	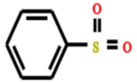	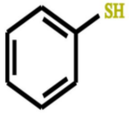	related fragments and losses [ClassyFire - Relevant terms - Substituents: nobenzenesulfonam Benzenesulfonyl group - Taxa: nobenzenesulfonam	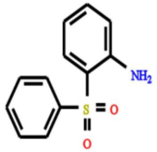	CSI: FingerID & MS2LDA
94	None	None	None	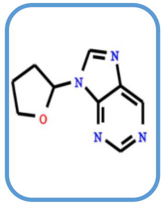	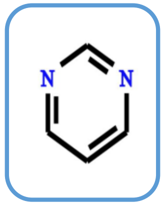	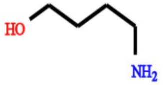	None	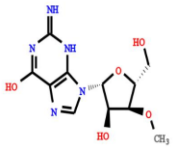	CSI: FingerID
95	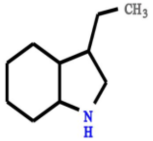	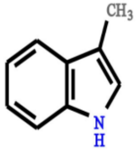	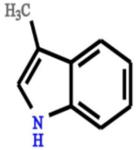	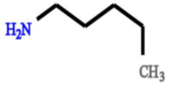	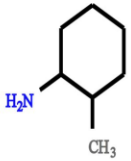	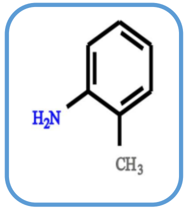	None	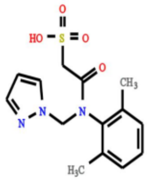	CSI: FingerID

96	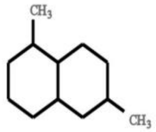	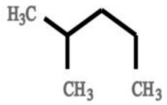	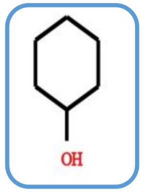	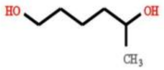	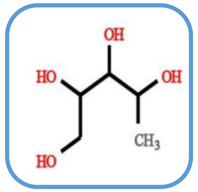	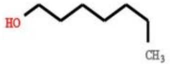	[illegible]yl-4-oxo-3,4-dihydro[illegible] substructure	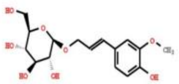	MESSAR & CSI: FingerID
97	None	None	None	None	None	None	None	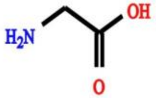	None
98	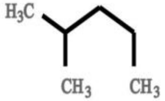	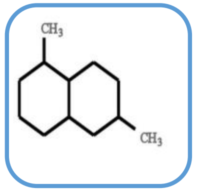	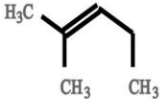	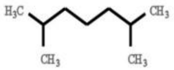	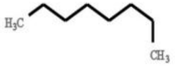	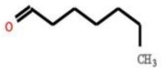	Water loss - indicative of a free hydroxyl group â€“ (in beer often seen in sugary structures)	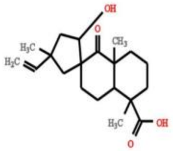	MESSAR
99	None	None	None	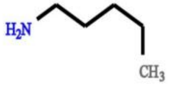	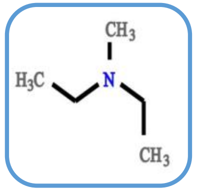	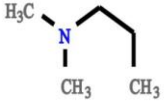	None	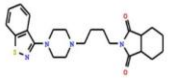	CSI: FingerID
100	None	None	None	None	None	None	None	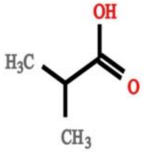	None

101	None	None	None	None	None	None	Amine loss - Indicative for free NH2 group in fragmented molecule	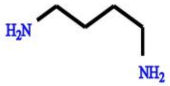	MS2LDA
102	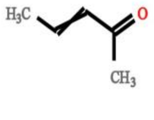	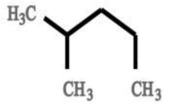	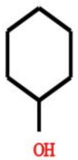	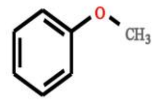	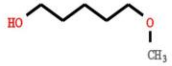	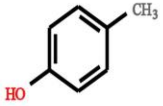	None	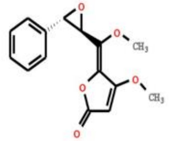	CSI: FingerID
103	None	None	None	None	None	None	None	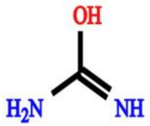	None
104	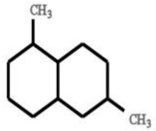	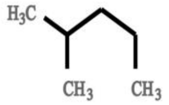	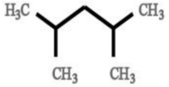	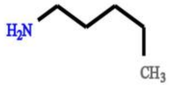	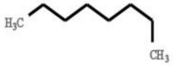	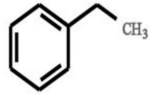	Fragments indicative for ferulic acid based substructure (MzCloud)	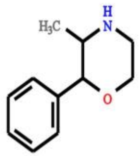	CSI: FingerID
105	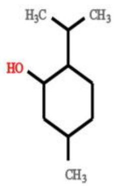	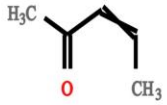	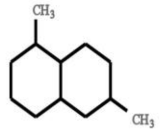	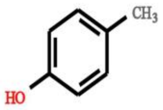	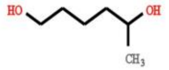	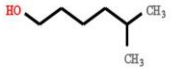	[illegible]phenyl)-2-oxo-2H-chr[illegible] related substructure (or isomeric variants)	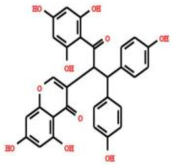	MESSAR & MS2LDA

106	None	None	None	None	None	None	None	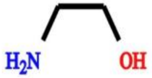	None
107	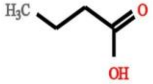	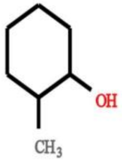	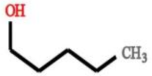	None	None	None	None	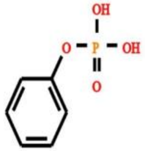	MESSAR
108	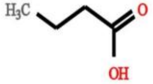	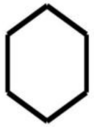	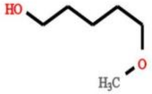	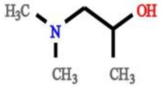	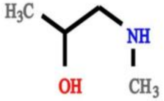	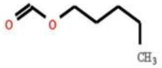	Sterone steroid related Mass2Motif	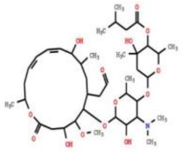	MESSAR
109	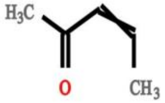	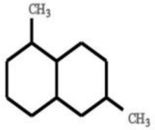	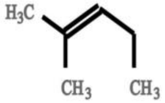	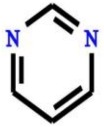	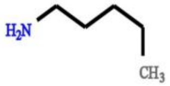	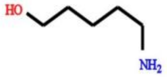	None	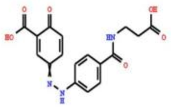	CSI: FingerID
110	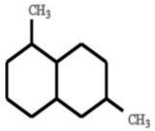	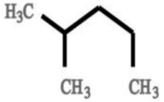	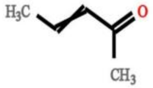	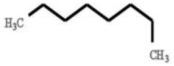	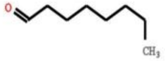	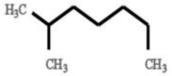	None	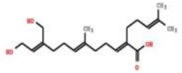	None

111	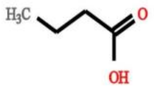	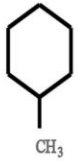	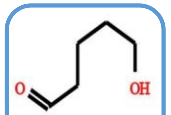	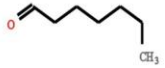	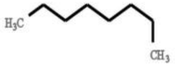	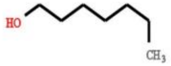	None	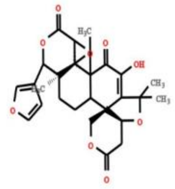	MESSAR
112	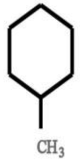	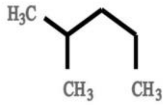	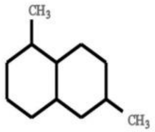	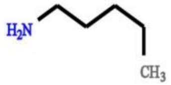	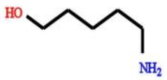	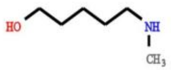	None	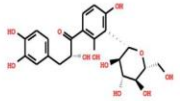	None
113	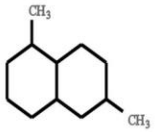	None	None	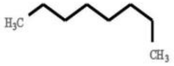	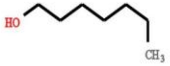	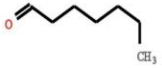	phthalate substructure	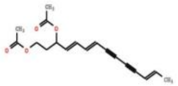	None
114	None	None	None	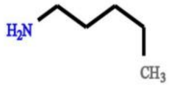	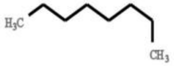	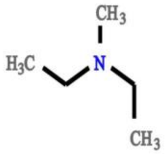	None	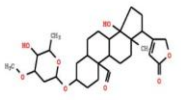	None
115	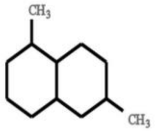	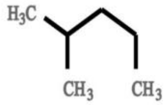	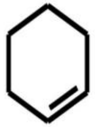	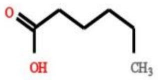	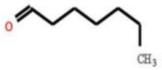	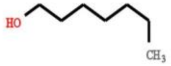	Double water loss i.e. 2*H20 â€“ Generic feature for metabolites containing several free OH groups attached to a aliphatic chain like [illegible]	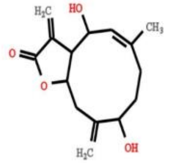	MS2LDA

116	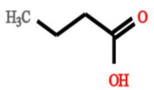	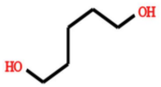	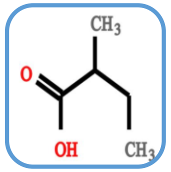	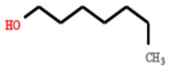	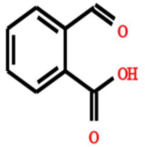	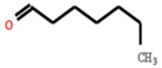	phthalate substructure	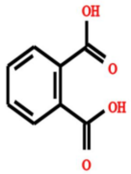	MESSAR & MS2LDA
117	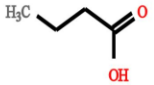	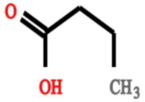	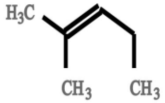	None	None	None	None	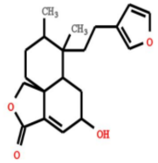	None
118	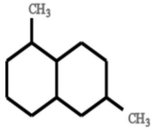	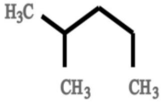	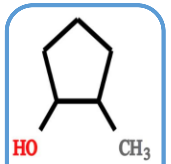	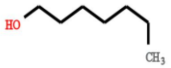	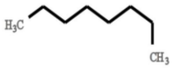	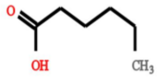	Double water loss i.e. 2*H20 â€“ Generic feature for metabolites containing several free OH groups attached to a aliphatic chain like [illegible]	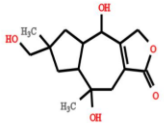	MESSAR
119	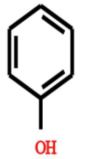	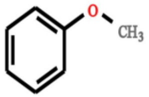	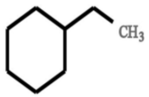	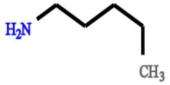	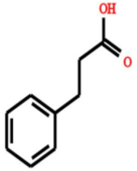	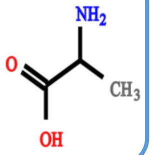	Fragment indicative for aromatic compounds related to methylbenzene substructure (C7H7 fragment)	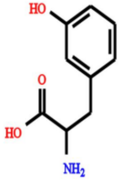	MESSAR & CSI: FingerID
120	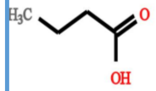	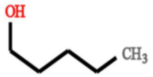	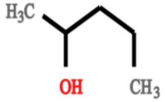	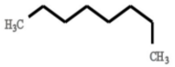	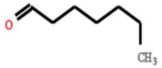	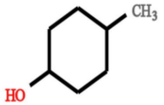	Fragments indicative for namic/hydroxycinnar acid substructure	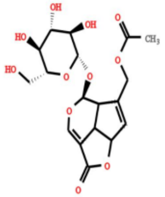	MESSAR

121	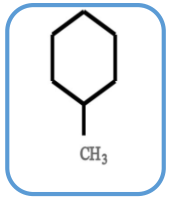	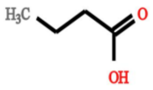	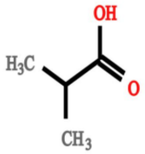	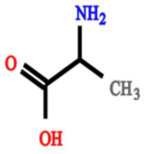	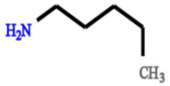	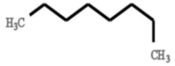	Loss of CH2O2 - indicative for underivatized carboxilic acid group	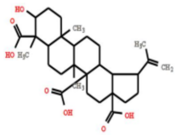	MESSAR
122	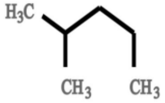	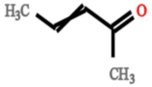	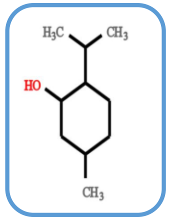	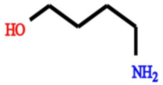	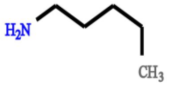	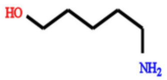	None	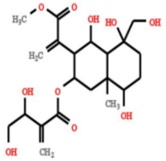	MESSAR
123	None	None	None	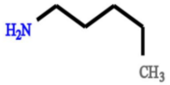	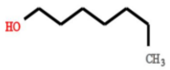	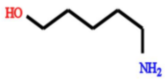	None	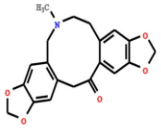	None
124	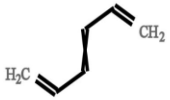	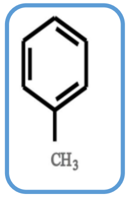	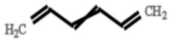	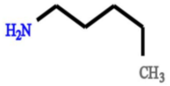	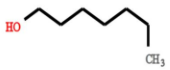	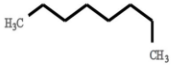	[illegible] loss i.e. 2*H20 â€“ Generic feature for metabolites containing several free OH groups attached to a aliphatic chain like [illegible]	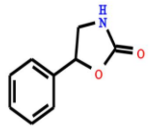	MESSAR
125	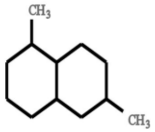	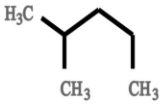	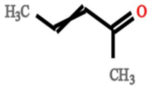	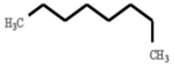	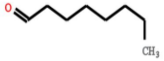	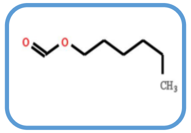	None	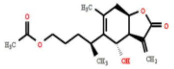	CSI: FingerID

126	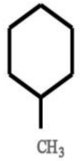	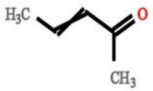	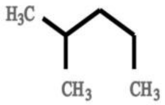	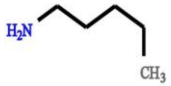	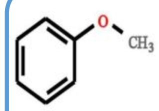	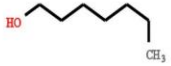	2-oxochromen-7-yl [mainly dimethylated] related substructure	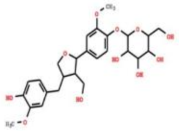	CSI: FingerID
127	None	None	None	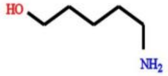	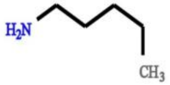	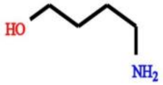	None	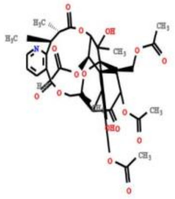	None
128	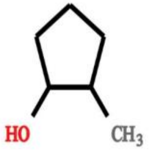	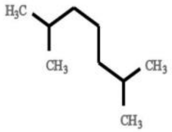	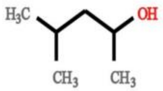	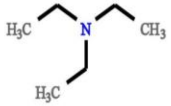	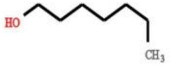	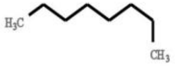	None	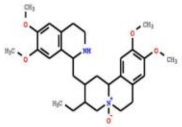	CSI: FingerID
129	None	None	None	None	None	None	None	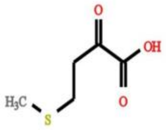	None
130	None	None	None	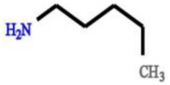	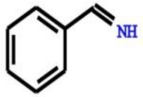	None	None	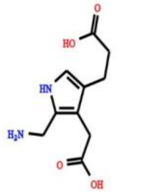	None

131	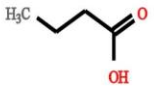	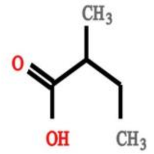	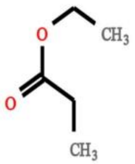	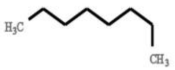	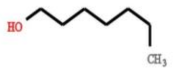	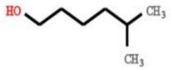	None	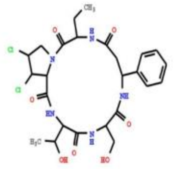	None
132	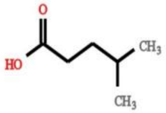	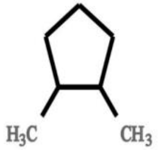	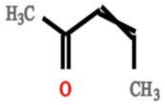	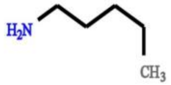	None	None	None	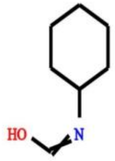	None
133	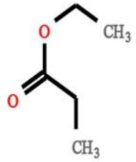	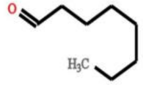	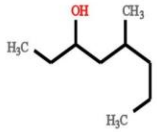	None	None	None	Amine loss - Indicative for free NH2 group in fragmented molecule	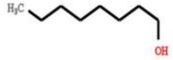	None
134	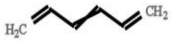	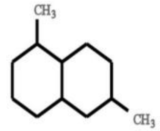	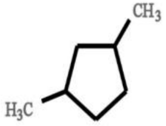	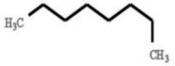	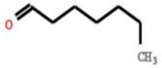	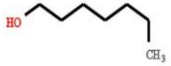	None	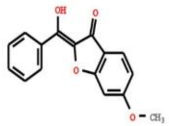	None
135	None	None	None	None	None	None	Fragments indicative of a glycosylation â€“ i.e. indicative for a sugar conjugation (in beer often related to glucose)	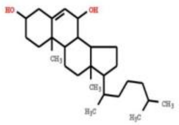	None

136	None	None	None	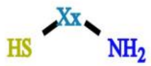	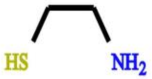	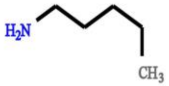	None	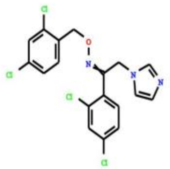	None
137	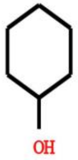	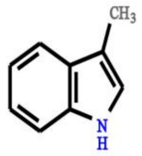	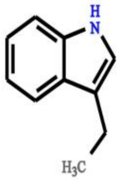	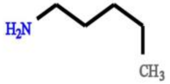	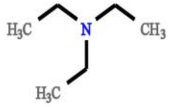	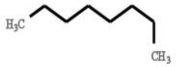	None	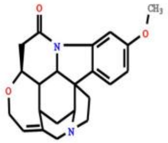	MESSAR
138	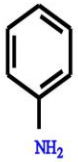	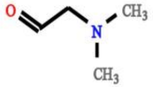	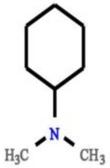	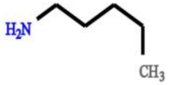	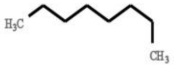	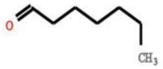	Small nitrogen containing fragment ion â€" often proline or ornithine derived â€" most abundant fragment in beer data	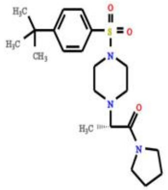	MESSAR
139	None	None	None	None	None	None	None	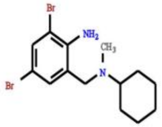	None
140	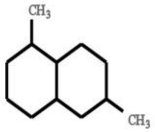	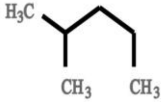	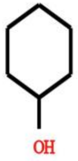	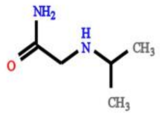	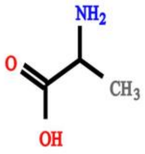	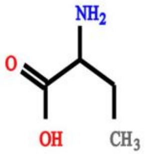	[illegible]ino)methyl]cyclohexa[illegible] acid related Mass2Motif (losses)	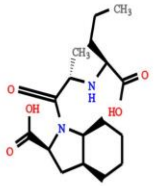	MESSAR & CSI: FingerID

141	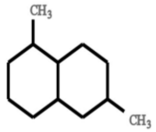	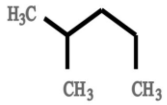	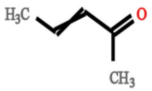	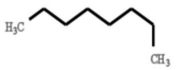	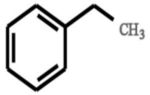	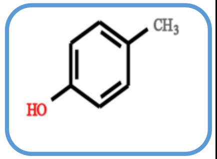	None	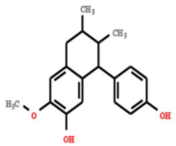	CSI: FingerID
142	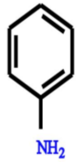	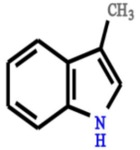	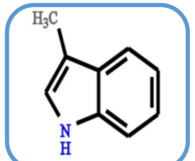	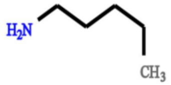	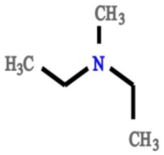	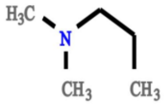	None	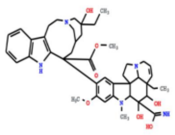	MESSAR
143	None	None	None	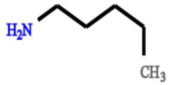	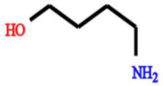	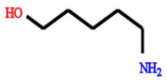	None	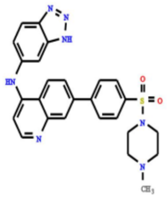	None
144	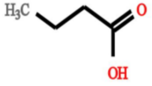	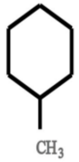	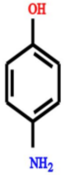	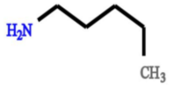	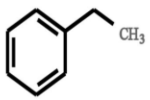	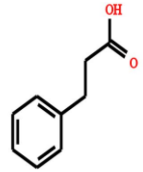	CO loss - indicative for presence of etone/aldehyde/lactor group (C=O)	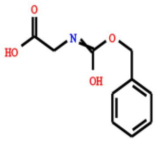	CSI: FingerID
145	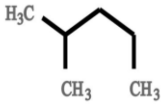	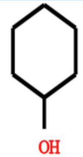	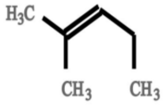	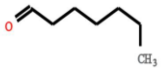	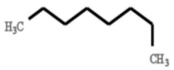	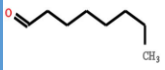	None	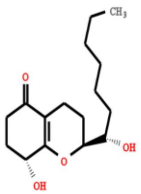	MESSAR & CSI: FingerID

146	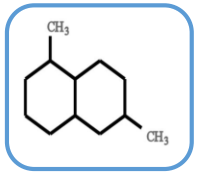	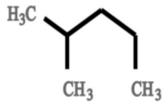	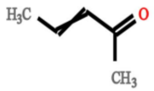	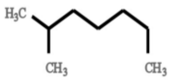	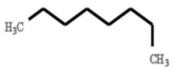	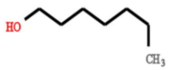	ydroxy-Pregnenolon related fragments (not very specific - indicative for presence of steroid backbone)	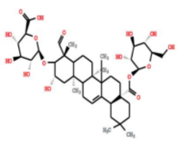	MESSAR & MS2LDA
147	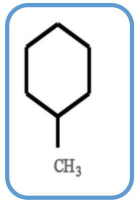	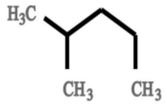	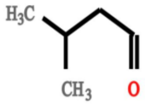	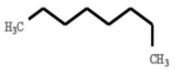	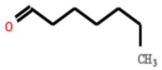	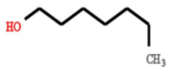	None	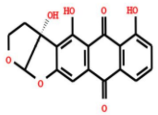	MESSAR
148	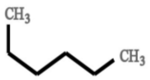	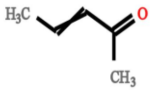	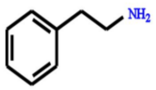	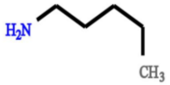	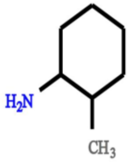	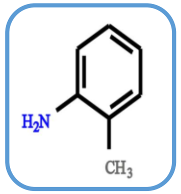	Indole substructure	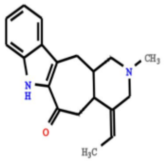	CSI: FingerID & MS2LDA
149	None	None	None	None	None	None	Loss possibly indicative of carboxylic acid group with 1-carbon attached.	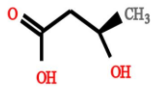	MS2LDA
150	None	None	None	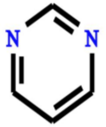	None	None	None	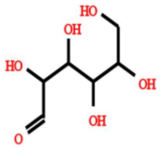	None

151	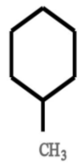	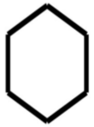	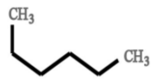	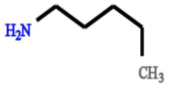	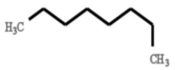	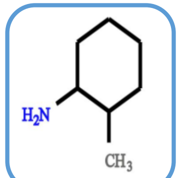	etrahydroisoquinoline substructure	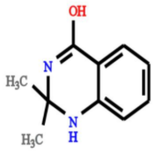	CSI: FingerID & MS2LDA
152	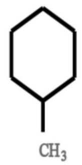	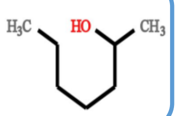	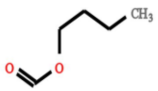	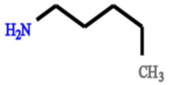	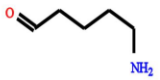	None	Fragment ions indicative for C6H12NO substructure (in beer related to N-acetylputrescine - MzCloud)	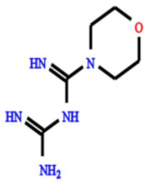	MESSAR & MS2LDA
153	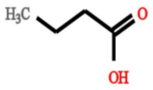	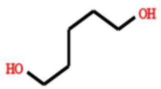	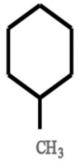	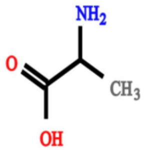	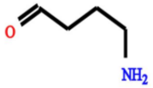	None	Amine loss - Indicative for free NH2 group in fragmented molecule	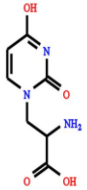	CSI: FingerID & MS2LDA
154	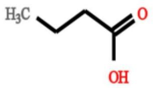	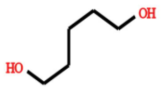	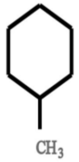	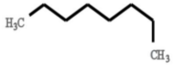	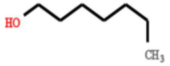	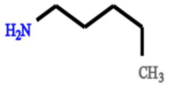	None	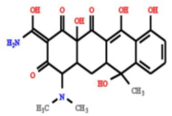	None
155	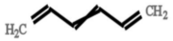	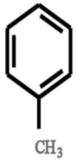	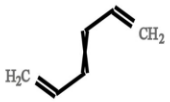	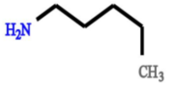	None	None	None	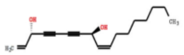	None

156	None	None	None	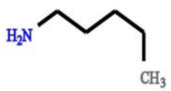	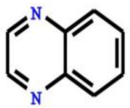	None	None	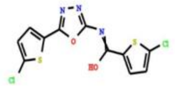	None
157	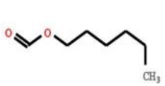	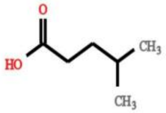	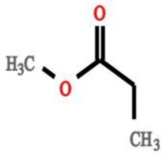	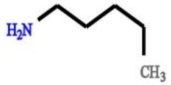	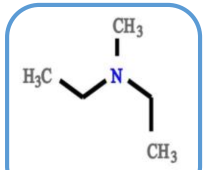	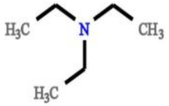	None	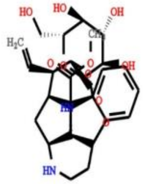	MESSAR & CSI: FingerID
158	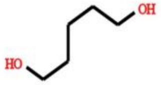	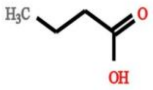	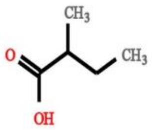	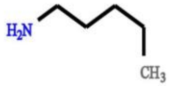	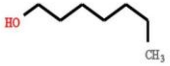	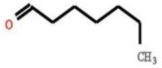	None	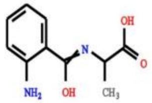	MESSAR & CSI: FingerID
159	None	None	None	None	None	None	None	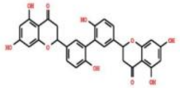	None
160	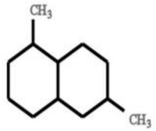	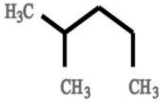	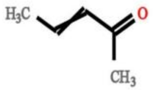	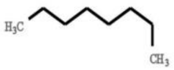	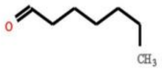	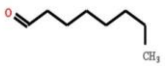	[illegible]ydroxy-Pregnenolon[illegible] related fragments (not very specific - indicative for presence of steroid backbone)	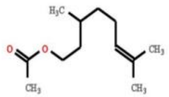	MESSAR

161	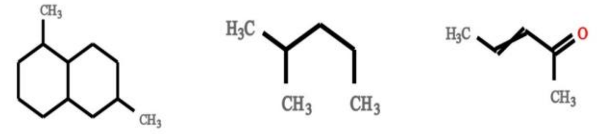	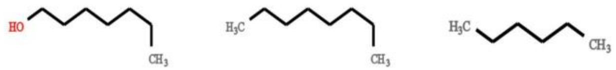	None	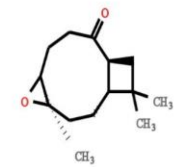	None
162	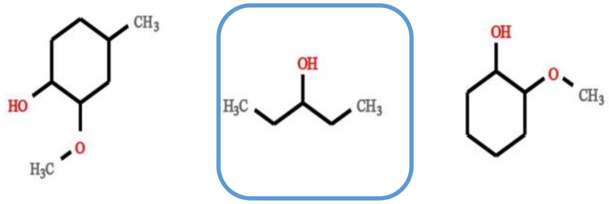	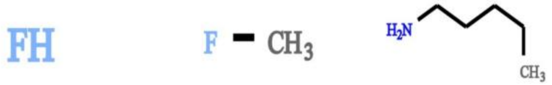	None	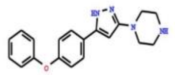	MESSAR
163	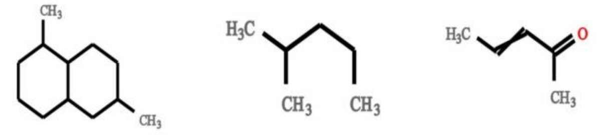	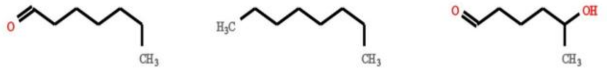	Pregn-4 or 5-ene-3-dione substructure	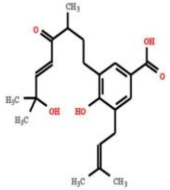	None
164	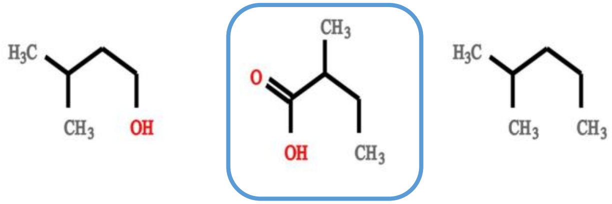	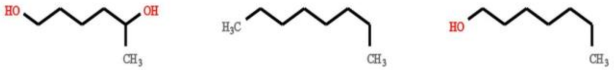	None	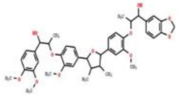	MESSAR
165	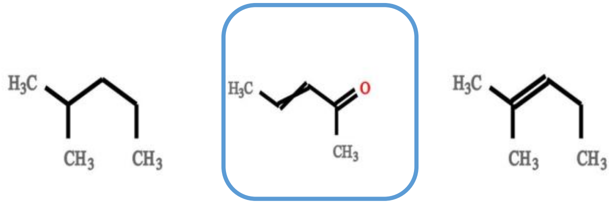	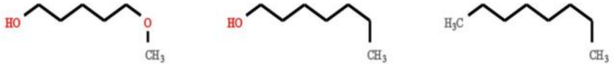	None	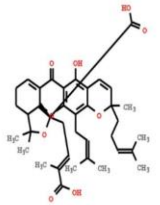	MESSAR

166	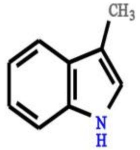	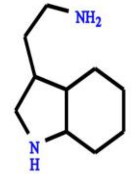	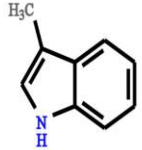	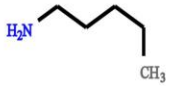	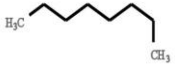	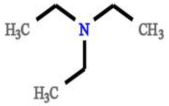	None	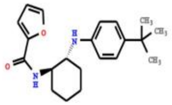	None
167	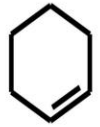	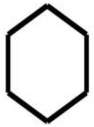	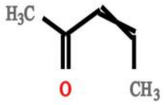	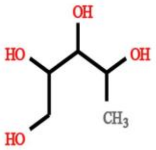	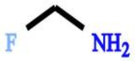	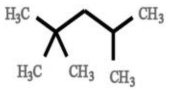	None	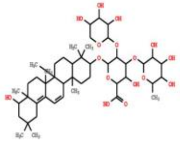	MESSAR
168	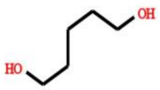	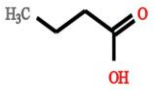	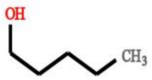	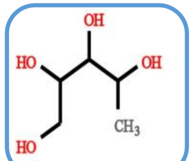	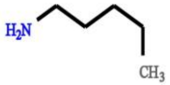	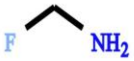	None	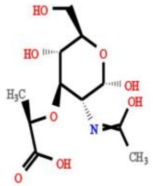	MESSAR & CSI: FingerID
169	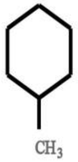	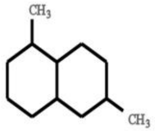	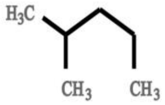	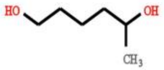	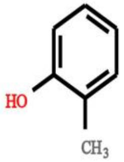	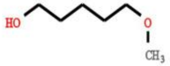	[hexose-H2O] â€“ indication of hexose conjugation (for example glucose) [ClassyFire - Relevant terms - Substituents: Hexose monosaccharide	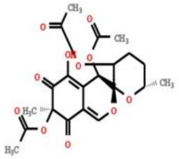	MS2LDA
170	None	None	None	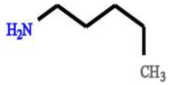	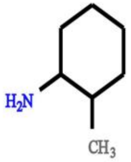	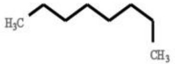	None	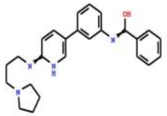	CSI: FingerID

171	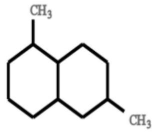	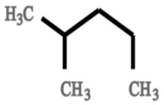	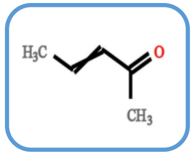	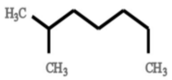	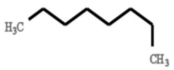	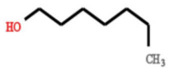	Steroid core related (C18H21 and smaller fragments thereof - with C12H13, C11H11, and C11H13 most probable)	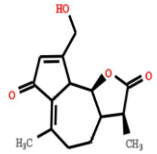	MESSAR
172	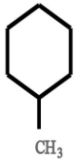	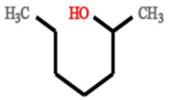	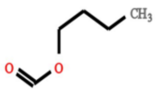	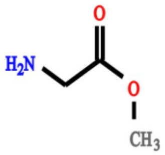	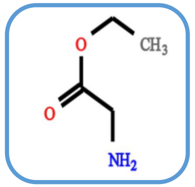	None	Fragments indicative of a glycosylation â€“ i.e. indicative for a sugar conjugation (in beer often related to glucose)	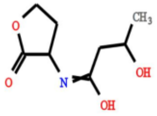	CSI: FingerID
173	None	None	None	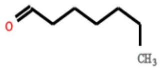	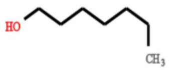	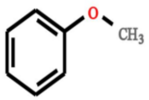	None	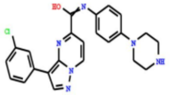	None
174	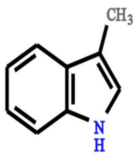	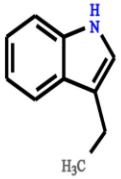	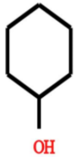	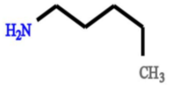	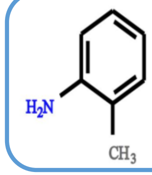	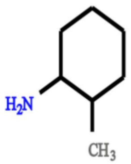	Indole substructure	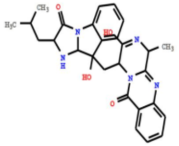	CSI: FingerID
175	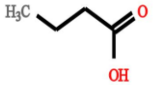	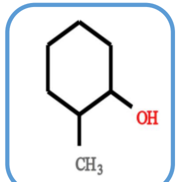	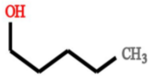	None	None	None	henyl)-2-oxo-2H-chr related substructure (or isomeric variants)	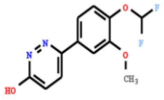	MESSAR & MS2LDA

176	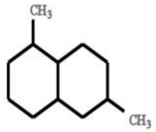	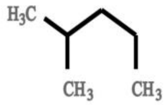	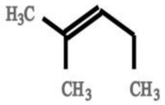	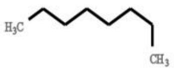	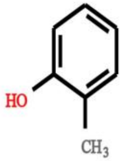	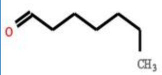	Fragments indicative for namic/hydroxycinnar acid substructure	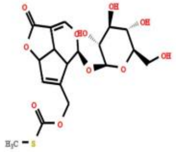	CSI: FingerID
177	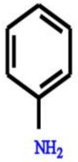	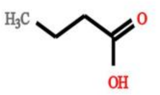	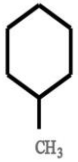	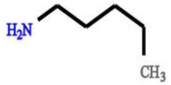	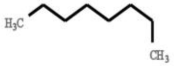	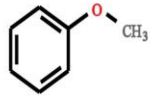	Indole substructure	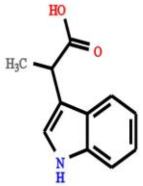	MESSAR & MS2LDA
178	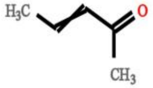	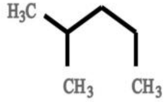	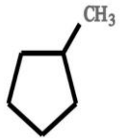	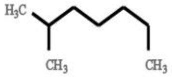	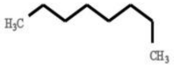	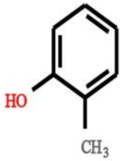	CO loss - indicative for presence of etone/aldehyde/lactor group (C=O)	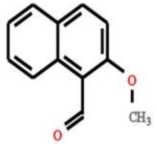	CSI: FingerID
179	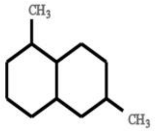	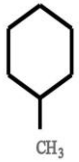	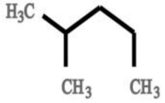	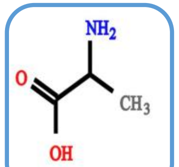	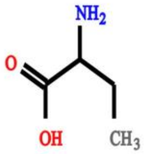	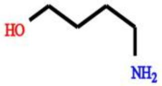	None	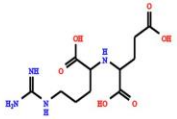	CSI: FingerID
180	None	None	None	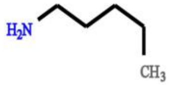	None	None	None	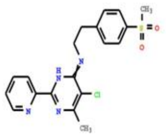	None

181	None	None	None	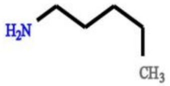	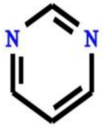	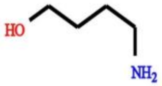	None	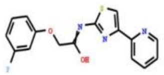	None
182	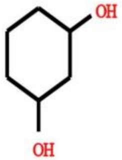	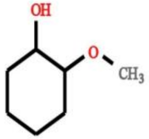	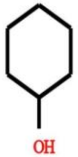	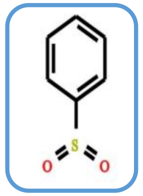	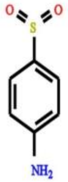	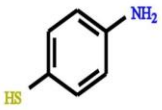	None	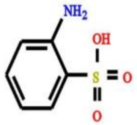	CSI: FingerID
183	None	None	None	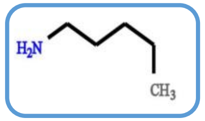	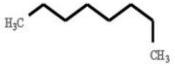	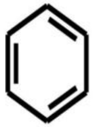	None	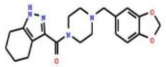	CSI: FingerID
184	None	None	None	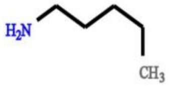	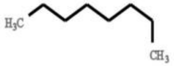	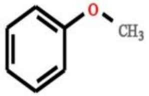	None	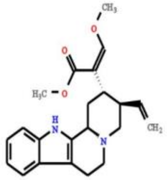	None
185	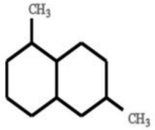	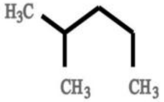	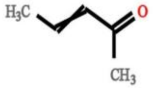	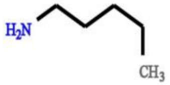	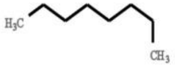	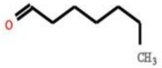	Sterone related	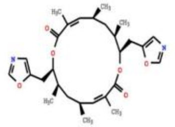	None
